# The mechanisms of action of metformin on head and neck cancer in the pre-clinical setting: a scoping review

**DOI:** 10.3389/fonc.2024.1358854

**Published:** 2024-02-22

**Authors:** Lucy Huang, Charmaine M. Woods, Nuwan Dharmawardana, Michael Z. Michael, Eng Hooi Ooi

**Affiliations:** ^1^ College of Medicine and Public Health, Flinders University, Adelaide, SA, Australia; ^2^ Department of Otolaryngology Head and Neck Surgery, Flinders Medical Centre, Adelaide, SA, Australia; ^3^ Department of Gastroenterology and Hepatology, Flinders Medical Centre, Adelaide, SA, Australia

**Keywords:** metformin, head and neck cancer, squamous cell carcinoma, cell lines, xenograft

## Abstract

This scoping review identifies the mechanistic pathways of metformin when used to treat head and neck cancer cells, in the pre-clinical setting. Understanding the underlying mechanisms will inform future experimental designs exploring metformin as a potential adjuvant for head and neck cancer. This scoping review was conducted according to the Joanna-Briggs Institute framework. A structured search identified 1288 studies, of which 52 studies fulfilled the eligibility screen. The studies are presented in themes addressing hallmarks of cancer. Most of the studies demonstrated encouraging anti-proliferative effects *in vitro* and reduced tumor weight and volume in animal models. However, a few studies have cautioned the use of metformin which supported cancer cell growth under certain conditions.

## 1 Introduction

Head and neck squamous cell carcinoma (HNSCC) is the 6^th^ most commonly diagnosed cancer worldwide (1), with an overall five year survival of only 72% (2). The key risk factors for developing HNSCC are consumption of carcinogens (tobacco, betel quid, alcohol) and the oncogenic human papillomavirus (HPV) (3). The management of HNSCC involves a multidisciplinary approach comprising surgical resection, radiotherapy and chemotherapy (3). During the last few decades, there has been little advancement in the chemotherapeutic options available (4).

HNSCC and its treatment(s) have significant consequences on patients’ daily functions and quality of life. Despite recent advances in modern medicine, there remains a high risk of local recurrence and distant metastases (5). Treatment options for local recurrences are generally limited to salvage surgery with minimal improvement in overall survival rates (6, 7). There is an urgent need for novel solutions to improve treatment opportunities for patients with head and neck cancer.

Drug repurposing is the use of currently approved drugs for an alternative indication (8), often reducing costs associated with drug development and the time to clinical translation (9). Metformin is an oral biguanide derived from the French lilac, *Galega officinalis*, and is widely used as the first-line treatment for type 2 diabetes mellitus. Other indications for metformin include polycystic ovary disease, obesity and gestational diabetes, thereby demonstrating its safety profile in a wide range of patients (10). The concept of metformin having anti-tumor properties was first introduced by Dilman in the early 1970s, using a derivative of metformin, phenformin, to reduce tumor formation (11, 12). This concept was revisited in 2005 following an epidemiological study that suggested patients with type 2 diabetes taking metformin have a reduced risk of cancer (13). This has since stimulated significant interest in the potential of using metformin as a treatment for cancer.

Metformin has been described to indirectly activate AMP-activated protein kinase (AMPK) in the liver to reduce blood glucose levels (14). AMPK is also described as a metabolic tumor suppressor in cancer development (15). It has therefore been hypothesized that metformin also acts directly on cancer cells by activating the AMPK pathway (16, 17). However, there is increasing evidence of alternative mechanistic pathways which are both dependent and independent of AMPK. A systematic review on this topic was conducted in 2015 including 11 studies (18). Since then, there have been an additional 41 studies published on the action of metformin in HNSCC, requiring an updated review. Scoping reviews have been designed to systematically map the available evidence and therefore suitable to address the aim of this study (19). This scoping review aims to collate the reported mechanisms of action of metformin in HNSCC in the pre-clinical setting. Mapping the identified cellular pathways will help inform study designs when using metformin, either alone or as a combination therapy, for the treatment of head and neck cancer.

## 2 Methods

This scoping review followed the methodology published by the Joanna Briggs Institute (20).

### 2.1 Search strategy

This search aims to identify published literature. To identify keywords for the search strategy, an initial search of MEDLINE and CINAHL using keywords (Head and neck cancer) AND (Metformin) was conducted to screen for articles and develop full search terms through a screen of the title, abstract and indexed terms in consultation with a Flinders University librarian in March 2021. An updated search was conducted in May 2023. Details on the search terms are provided in **Supplementary Table 1**. The databases subsequently searched include MEDLINE, CINAHL, Embase, SCOPUS, and Cochrane. Reference lists of included studies were reviewed to identify additional studies.

### 2.2 Inclusion criteria

This review systematically maps the available evidence from studies that used cell culture and animal models in the pre-clinical laboratory setting, specifically in HNSCC of mucosal origin: oral cavity, oropharynx, hypopharynx, and larynx. The studies are limited to English, with no limitation to the timeframe.

### 2.3 Exclusion criteria

The exclusion criteria are HNSCC of other subsites (salivary gland, sinonasal cavity, nasopharynx and cutaneous). Studies using results derived from reported contaminated cell lines (Hep-2, KB, Ca9-22) (21–24) were also excluded. One study using a syngeneic mouse model was also excluded as it utilized murine cancer cell lines (25). Clinical studies involving patients are beyond the scope of this review.

### 2.4 Study selection

**Figure 1:**
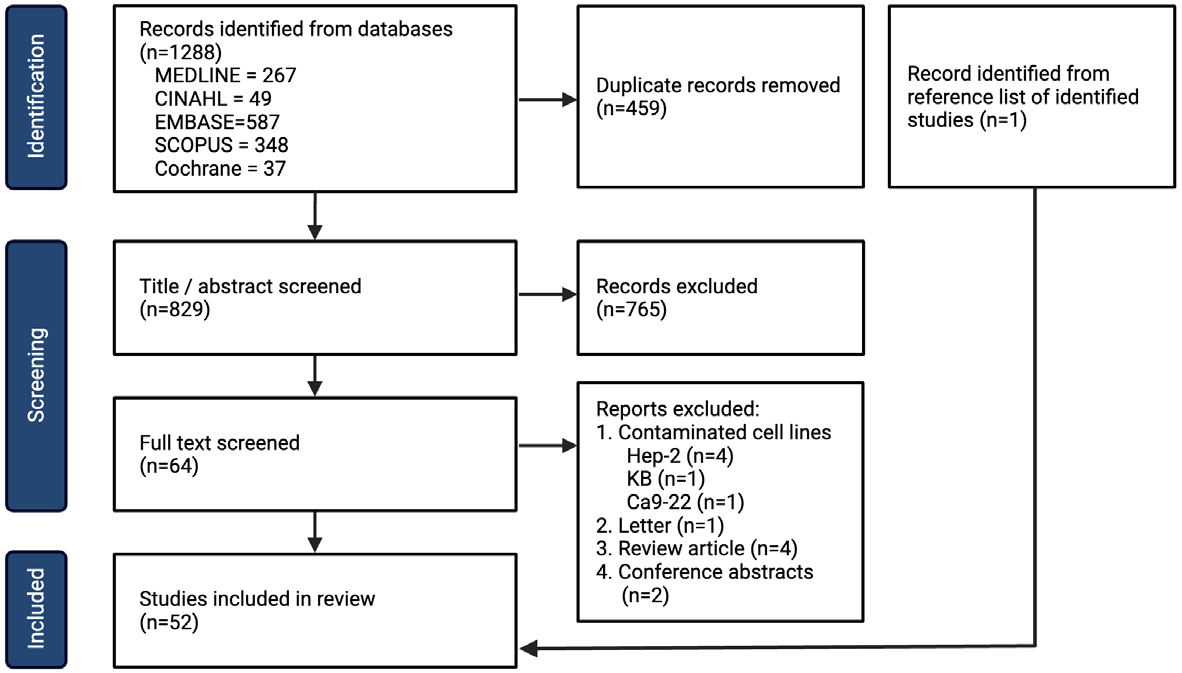
PRISMA flow chart for inclusion of studies.

Following the search, all identified studies were uploaded into Covidence systematic review software (Veritas Health Innovation, Melbourne, Australia) and duplicates were removed. Two independent reviewers (LH and ND) screened studies based on title and abstract assessment. Disagreements were resolved with a full-text review and a discussion amongst the two reviewers. Full-text review was then assessed against the inclusion and exclusion criteria. The reasons for exclusion following full-text review are listed in **Figure 1** (PRISMA 2020) (26).

### 2.5 Development of themes

Studies were discussed according to the hallmarks and enabling characteristics of cancer (27, 28). A few studies discussed properties of metformin that do not fall within the described hallmarks and will be discussed separately.

### 2.6 Data presentation

The described molecular pathways are compiled and illustrated using BioRender (BioRender.com).

## 3 Results

A total of 1288 studies were identified with 829 remaining after removal of duplicates, amongst which 52 studies met the inclusion criteria (**Figure 1**). The year, country and experimental designs of the identified studies are provided in **Supplementary Data 1** and **Supplementary Table 2**. There has been an increase in the number of studies published with a peak of 10 studies published in 2019. Geographically, China has published the greatest number of studies (n=26) since 2011. The experimental designs of the preclinical studies are listed in **Supplementary Table 3**. A summary table of the cell lines, their original anatomical subsites and the reported mutations are provided in **Supplementary Table 4**. 29 HNSCC cell lines were used, with the oral tongue being the most common subsite. 4 cell lines were reported to be HPV-positive. The studies of metformin as a single agent *in vitro* (n=44) are summarized in **Supplementary Table 5**, metformin in combination with another therapy *in vitro* (n=24) are summarized in **Supplementary Table 6**, and metformin use *in vivo* (n=25) are summarized in **Supplementary Table 7**. Metformin doses vary significantly from 10 μM (29) to 100 mM (30).

## 4 Discussion

In pre-clinical studies of mucosal HNSCC, metformin is reported to modulate several hallmarks of cancer (31), resulting in reduced cell growth *in vitro* and *in vivo.* Transcriptomic profiling identified differentially expressed genes following metformin treatment in HNSCC cell lines affecting all aspects of cancer development (32). Numerous mechanisms have been proposed and are summarized in this discussion.

### 4.1 Cellular uptake of metformin

Due to metformin’s hydrophilic properties, cellular uptake is dependent on active organic cation transporters (OCTs) (33). OCT3 is highly expressed in histological samples of dysplastic oral lesions and well-to-moderately differentiated HNSCC tumors but is significantly reduced in poorly differentiated tumors (34, 35). The viability of HN4 cell lines, which lack OCT3 expression, was not affected by metformin treatment (34). Further support was provided by the knockdown of OCT3 xenograft mouse models, which also demonstrated reduced activation of AMPK and increased mammalian target of rapamycin (mTOR) activity, negating the inhibitory effects of metformin (34, 36). These studies demonstrate that OCT3 is necessary for metformin to affect HNSCC cells. Therefore, cancer cells in poorly differentiated HNSCC, with low or no OCT3 expression, may not be affected by metformin. Future studies could investigate how HNSCC differentiation status and OCT3 expression may affect the response of metformin clinically.

### 4.2 Cellular metabolism

#### 4.2.1 Pyruvate metabolism

**Figure 2:**
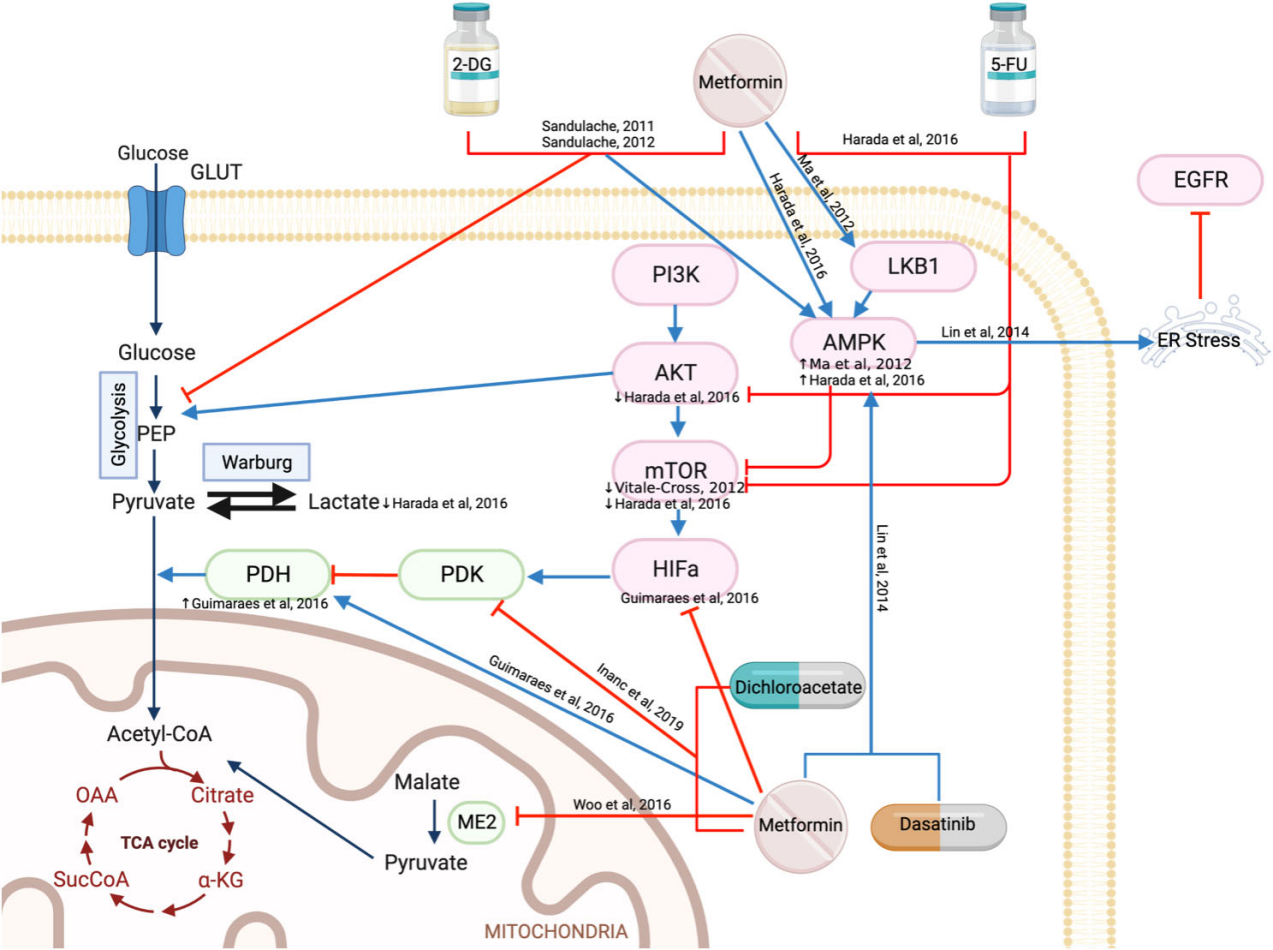
Metformin regulates mitochondrial energy metabolism.

The Warburg Effect, first discussed by Otto Warburg in 1956, is a metabolic shift in cancer cells that leads to the production of ATP via aerobic glycolysis, rather than oxidative phosphorylation, despite the presence of abundant oxygen (37). Many mechanisms contribute to this metabolic shift; with one being the oncogenic phosphoinositide-3-kinase (PI3K)/AKT/mTOR pathway, increasing glucose uptake and glycolysis (38, 39). Metformin targets the Warburg effect by activating AMPK to downregulate the mTOR pathway which in turn reduces HIF-1α, increases pyruvate dehydrogenase (PDH), and increases the conversion of pyruvate to acetyl-CoA rather than lactate (**Figure 2**). The chemotherapeutic agent, 5-fluorouracil (5-FU) has also been reported to inhibit AKT and mTOR, thereby reducing the downstream Warburg effect. The combination of metformin and 5-FU demonstrated a greater reduction in cell or tumor proliferation and an increase in apoptosis compared to single agents alone *in vitro* and *in vivo*, both acting on this mechanistic pathway (40).

Hypoxia-inducible factor (HIF)-1ɑ is activated in the hypoxic tumor environment, which in turn stimulates pyruvate dehydrogenase kinase (PDK) to inactivate pyruvate dehydrogenase (PDH) (41). This prevents the conversion of pyruvate to acetyl-CoA, instead promoting pyruvate conversion to lactate. As a result, lower PDH and higher HIF-1ɑ are present in patients with oral SCC compared to those with oral premalignant lesions (42). Metformin treatment reduced HIF-1ɑ, upregulated PDH mRNA and reduced heat shock protein 90 (HSP90), resulting in reduced cell proliferation and migration, increased apoptosis, and DNA fragmentation (42). Furthermore, metformin is reported to work synergistically with dichloroacetate (an inhibitor of PDK) in reducing cell viability (43) (**Figure 2**). While mechanisms were not explored, dichloroacetate is expected to prevent the inactivation of PDH and allow pyruvate to progress into the tricarboxylic acid cycle (**Figure 2**) (44).

Malic enzyme 2 catalyzes the conversion of malate to pyruvate by reducing NADH to NADPH within the mitochondria and plays a key role in redox balance and energy production (45). It modulates AMPK/AKT pathways, p53 function, cellular differentiation, glutamine oxidation and reactive oxygen species (ROS) production to promote the survival of cancer cells (45). Analysis of The Cancer Genome Atlas (TCGA) data demonstrates that overexpression of malic enzyme 2 in HNSCC is linked with lower overall survival (46). Metformin reduced malic enzyme 2 in both wild-type p53 (HN30) and mutant p53 (HN31) cell lines, which activate both p21 and ROS to induce senescence (46) (**Figure 2**).

#### 4.2.2 Glucose metabolism

**Figure 3:**
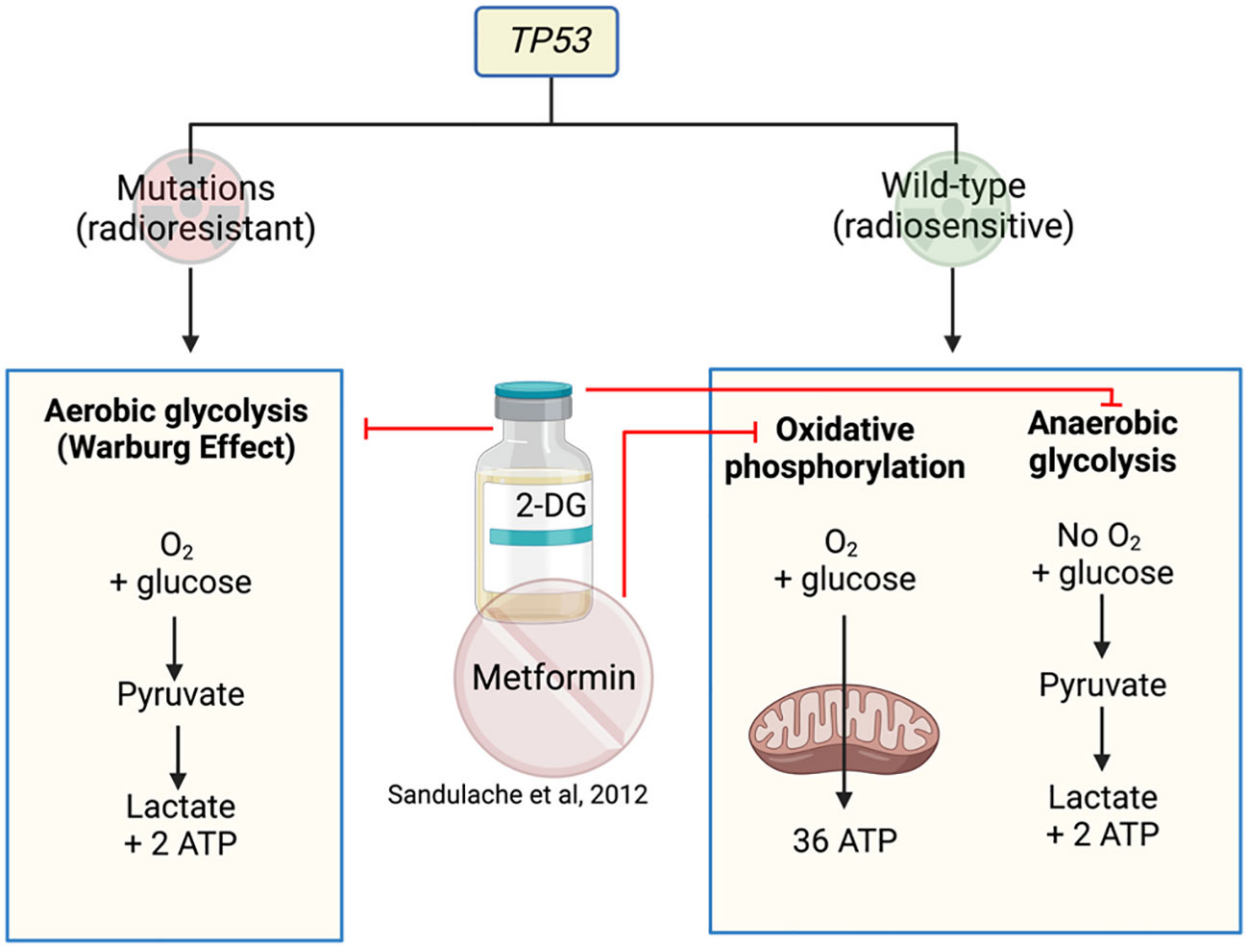
Metformin and 2-DG target metabolic pathways in cell lines with or without *TP53* mutations.

*TP53* is a gene that encodes for the p53 protein and is frequently mutated in HNSCC. HNSCC cells are highly dependent on glucose for survival with mutated (mut) *TP53* preferencing glycolysis over mitochondrial respiration (47, 48). 2-deoxy-D-glucose (2-DG) is a glucose analogue that competitively inhibits glucose uptake and prevents further glycolysis (49). Therefore, targeting glucose metabolism with 2-DG, in combination with metformin in the isogenic cell lines HN30 and HN31, reduced overall cell numbers (47) (**Figure 3**). A follow-up study performed by the same investigators analyzed the differing responses to 2-DG and radiation in cells with mut*TP53* compared to wt*TP53* (48) to find mut*TP53* cell lines more radioresistant than wt*TP53.* Cell lines with mut*TP53* were found to have less mitochondrial reserve, with a greater dependence on glycolysis, thus rendering them more susceptible to glycolysis inhibitor 2-DG which increased their radiosensitivity. In contrast, cells with preserved wt*TP53* prefer mitochondrial oxidative phosphorylation resulting in relative insensitivity to glycolytic inhibition by 2-DG. Metformin can inhibit mitochondrial respiration in both mut*TP53* and wt*TP53*, reducing oxygen consumption rates, leading to a compensational increase in glycolytic activity (48). As a result, the combination of metformin and 2-DG significantly impacts on the glycolytic activity in wt*TP53* cells that are less sensitive to 2-DG treatment. Depending on the *TP53* mutational status, radio sensitization could occur using 2-DG with or without metformin (48) (**Figure 3**). This appears paradoxical, as the study describes using combination drugs (2-DG and metformin) in wt*TP53* cells which are more radiosensitive but only using one drug (2-DG) for mut*TP53* cell lines. However, it is an interesting concept to use 2-DG and metformin in combination to target the two metabolic pathways.

#### 4.2.3 Glutamate metabolism

**Figure 4:**
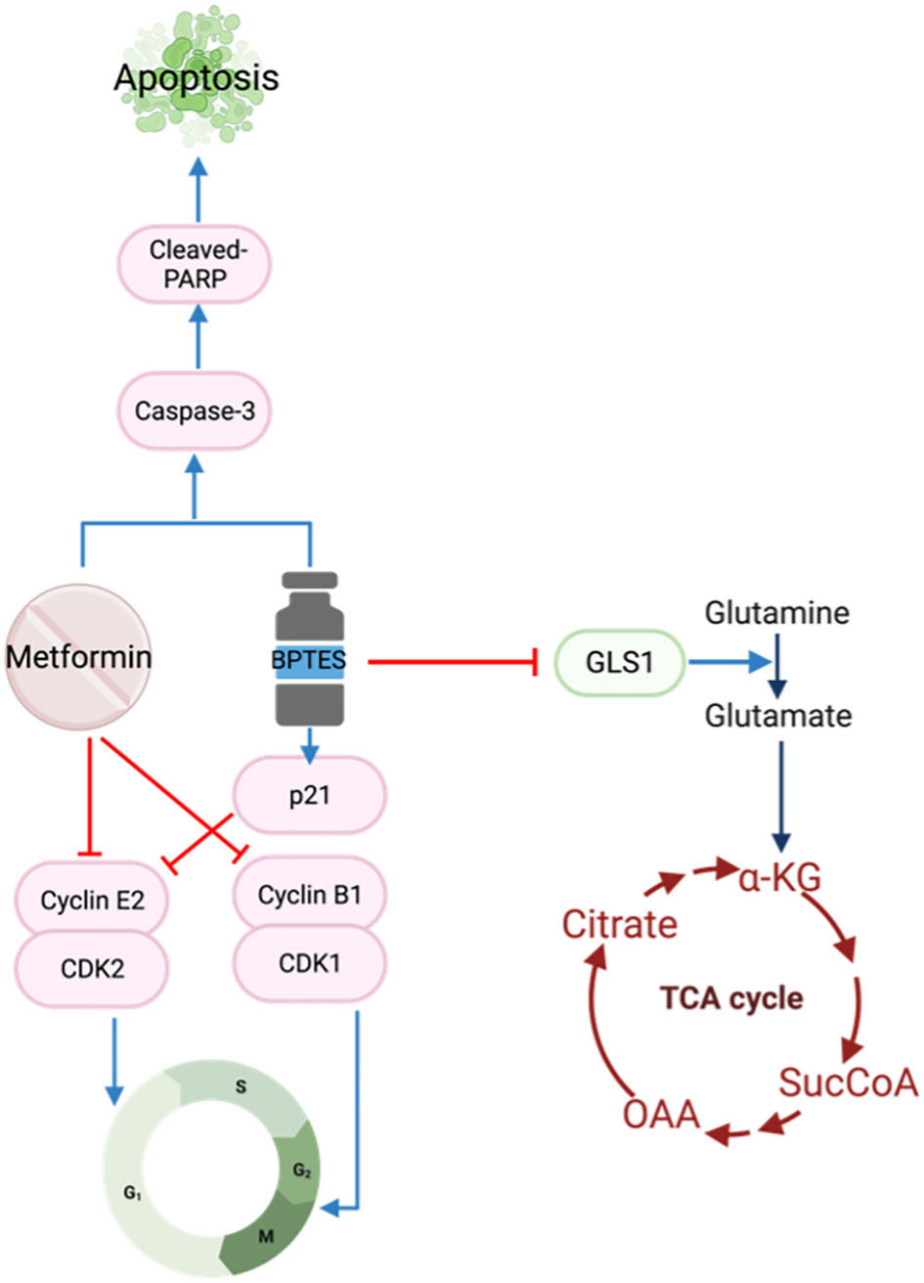
Metformin and BPTES target both cell cycle regulatory proteins and apoptotic pathways.

Glutamine is another important fuel for the proliferation and survival of HNSCC cells (50). Glutaminase 1 (GLS1) is a key enzyme converting glutamine to glutamate which is subsequently transformed to ɑ-ketoglutarate for the Krebs cycle (**Figure 2**). The TCGA database reports GLS1 to be highly expressed in HNSCC cell lines (51), stimulating an interest in using the selective glutaminase inhibitor bis-2-(5-phenylacetamido-1,3,4-thiadiazol-2-yl)ethyl sulfide (BPTES) in cancer treatment. The increase in GLS1 expression suggests altered glutamine metabolism in HNSCC cells allows for glutamine-dependent growth. The combination of BPTES and metformin reduced cell growth, cell viability and increased apoptosis by targeting apoptosis and cell cycle pathways (51). To induce cell cycle arrest, BPTES increased p21 while metformin reduced Cyclin E2 and cyclin B1/CDK1 complexes. Both BPTES and metformin were able to trigger the Caspase 3/PARP cascade to induce apoptosis (51) (**Figure 4**).

### 4.3 Proliferative signals

#### 4.3.1 AMPK/mTOR pathway

AMPK is a protein kinase that is essential in the restoration of energy homeostasis in response to metabolic stress with mTOR being one of its downstream targets (52). Metformin alone was demonstrated to reduce viability, proliferation, and colony formation *in vitro* (36, 53–56) and reduce tumor volume and weight *in vivo* (36, 55, 56). Treatment of HNSCC cell lines with metformin resulted in G0/G1 cell cycle arrest, activation of AMPK, and reduction in mTOR, p-S6K, p4EBP and p70S6K (36, 55–57) (**Figure 2**).

Dasatinib, a kinase inhibitor used for the treatment of chronic myeloid leukemia, activates AMPK by reducing ATP through the inactivation of ERK. The activation of AMPK by metformin goes on to induce endoplasmic reticulum stress which degrades EGFR through the c-Cbl lysosome pathway, resulting in apoptosis (58) (**Figure 2**). With both dasatinib and metformin activating AMPK, synergistically they reduce cell viability, increase apoptosis *in vitro* and reduce tumor volume in a HNSCC xenograft model (58).

#### 4.3.2 Cell cycle arrest

**Figure 5:**
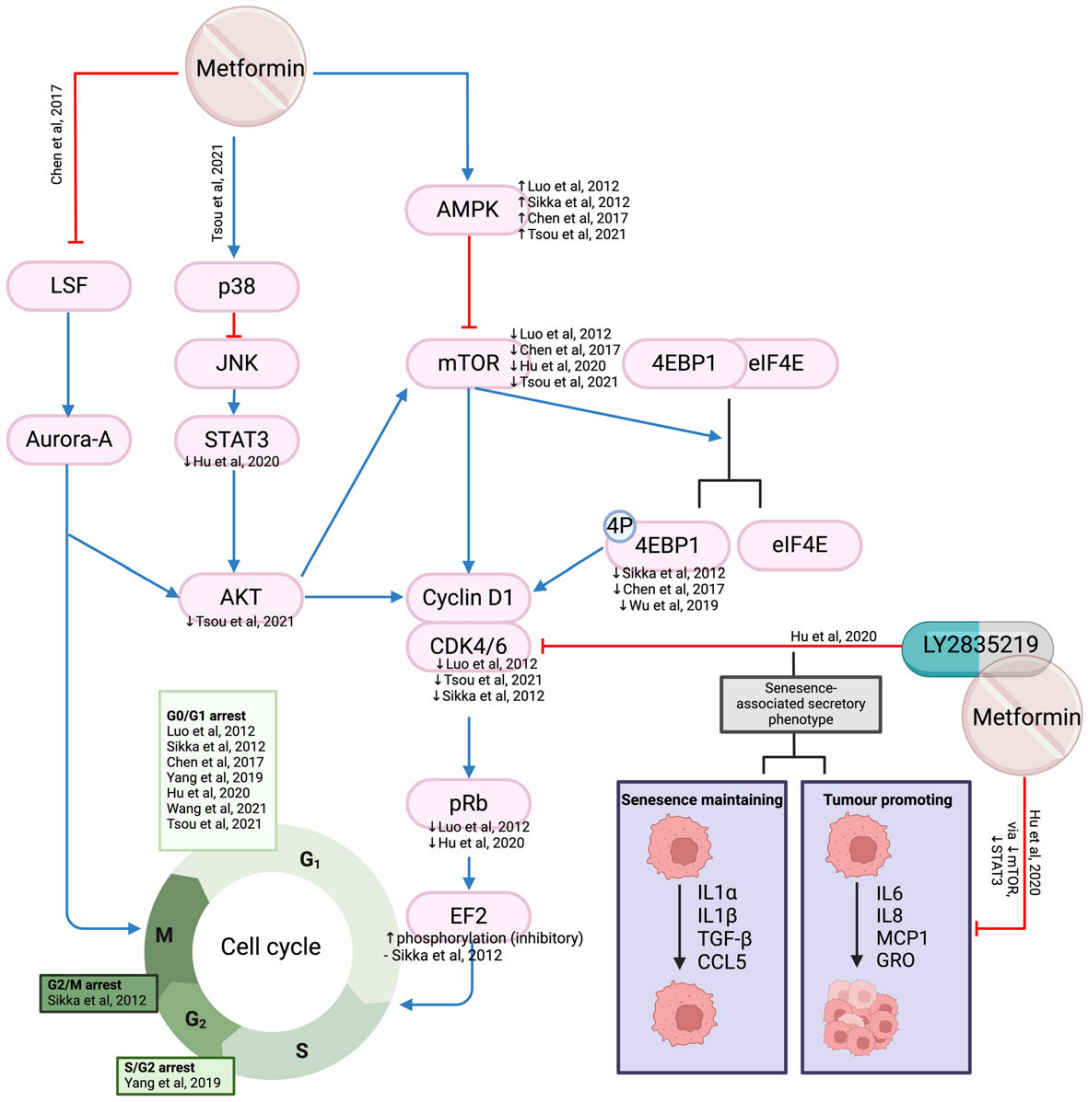
The mechanisms of action of metformin affecting the cell cycle in HNSCC. The regulation of proteins (marked as up or downward arrows) in response to metformin is referenced accordingly.

Cell cycle dysregulation in cancer cells can result in uncontrolled division (59). The TCGA data showed overexpression of cell cycle regulators cyclin D1, cyclin-dependent kinase (CDK) 4 and CDK6 in HNSCC, which are associated with poor survival (60). Normal cell cycle progression from G1 to S-phase requires Cyclin D1, CDK4, and CDK6 to phosphorylate retinoblastoma protein (pRb) and release transcription factor elongation factor (EF) 2 (61). Downstream targets of mTOR, cyclin D1 and associated CDK4, and CDK6, are downregulated due to metformin treatment (55, 62, 63) (**Figure 5**). The level of CDK inhibitors (p21 and p27) increased in some studies (63, 64) but not in others (55). Metformin also activates p38, which inhibits JNK/STAT 3/AKT with subsequent reduction in cyclin D1 mRNA and protein levels (62) (**Figure 5**). Additionally, Sikka et al. proposed that metformin inhibits protein translation via inhibition of translation initiation protein 4E-BP1 and EF2 through upstream regulator AMPK (64), subsequently reducing expression of cyclin D1, cyclin E, CDK2, and CDK4, resulting in cell cycle arrest (64) (**Figure 5**).

Chen et al. described metformin inhibiting Aurora A through Late SV40 Factor (LSF) (65) (**Figure 5**). Aurora-A plays an important role in the progression of G2/M transition and over-expression has been found to correlate with advanced TNM staging and poorer prognosis (66). Interestingly, Chen reported no change in cell cycle distribution despite a reduction in cell viability, colony formation and the ability to migrate or invade. This is in contrast to other studies where both metformin (55, 60, 62–64) and Aurora-A inhibitors (67) resulted in cell cycle arrest.

### 4.4 Growth suppressors

#### 4.4.1 Increase radiosensitivity in *TP53* mutant cells by increasing reactive oxygen species

**Figure 6:**
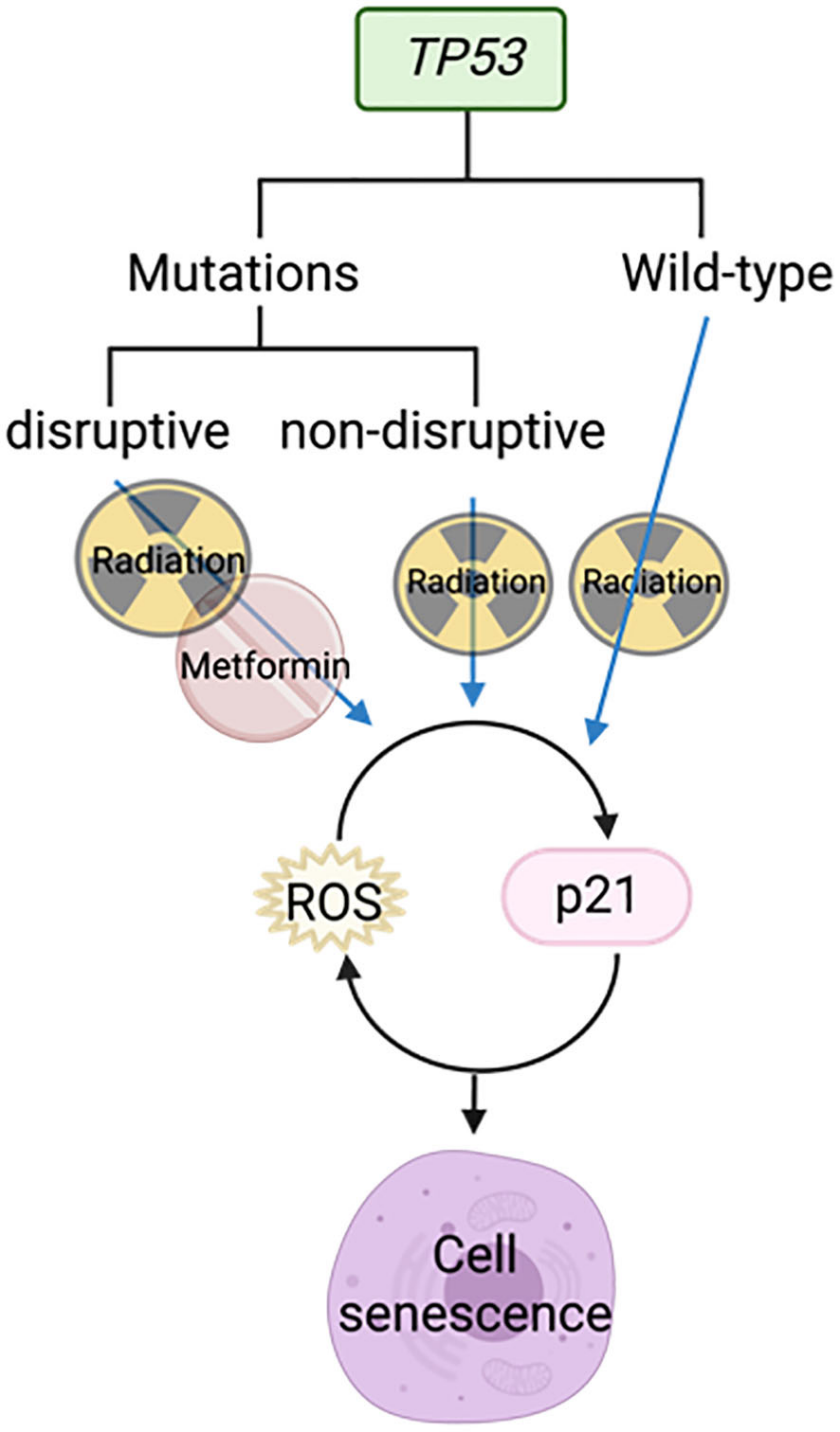
Metformin combined with radiotherapy targets disruptive *TP53* mutations to cause cell senescence.

Skinner et al. demonstrated using gene sequencing of 74 HNSCC samples that *TP53* mutations are predictive of increasing risk of locoregional recurrence, poorer overall survival, and the likelihood of radio resistance. “Disruptive” *TP53* mutations have a worse outcome than “wild-type” or “non-disruptive” mutations (68). Radiation was able to activate p21 and ROS in cell lines with wild-type and non-disruptive *TP53* mutations to induce senescence, but not those with disruptive mutations (**Figure 6**). However, the combination of metformin and radiotherapy increased radiosensitivity in cells with disruptive *TP53* mutations by increasing ROS (**Figure 6**). This was further supported by a reduction in clonogenic survival *in vitro* and reduced tumor growth *in vivo* when metformin and radiation were used in combination (68).

### 4.5 Reducing genome instability and mutation

#### 4.5.1 Reduce malignant transformation

Metformin was also shown to reduce the development of premalignant lesions in a 4-nitroquinoline 1-oxide (4NQO) rodent model. 4NQO is a water-soluble carcinogen that simulates the step-wise progression of human tobacco-related HNSCC in the oral cavity of the rodent (69). In mice treated with metformin (via intra-peritoneal injection), there was a reduction in the basal proliferation of the hyperplastic regions on the tongue and the number, size, and progression of the oral lesions to malignancy (39). This study supports the use of metformin as a chemoprevention measure for the development of premalignant lesions, which may be beneficial in high-risk patients with leukoplakia.

### 4.6 Senescent cells

Cell senescence is an outcome for many CDK inhibitors, aiming at cell cycle arrest, rendering them viable but unable to proliferate (60). Senescence-associated secretory phenotype (SASP) can act to either inhibit or promote cancer by secreting inflammatory cytokines. Metformin has been shown to act as a senostatic drug by reducing the release of tumor-promoting cytokines (interleukin (IL)6, IL8, monocyte chemoattractant protein, GRO-family chemokines) into the medium after treatment with CDK4/6 inhibitor LY2835219 (Ademaciclib) in oral squamous cell carcinoma (OSCC) cell lines, without changing senescence-inducing cytokines (IL1α, IL1β, transforming growth factor (TGF)-β, chemokine ligand 5) via the mTOR/STAT3 and IL6/STAT3 pathways (60) (**Figure 5**). The addition of metformin to LY2835219 did not change the proportion of senescent cells but changed the cytokines secreted by these cells. This study demonstrated the mechanisms that allow metformin and CDK inhibitors to work together to promote cell senescence.

### 4.7 Cell death

The evasion of cell death is a hallmark of cancer (70). In some instances, metformin induced apoptosis in HNSCC cells (54, 55). Anti-apoptotic proteins Bcl-2 and Bcl-xL were down-regulated (62) while pro-apoptotic protein Bax was up-regulated (55, 63). The combination of metformin and histone deacetylase inhibitor 4SC-202 increased Bax, p53 and intrinsic apoptosis (cleaved caspase-9, cleaved caspase-3, cleaved- PARP), but not extrinsic apoptosis marker caspase-8 (71). This pro-apoptotic characteristic has been validated *in vivo* with increased apoptotic tumor cells detected with TUNEL staining (55, 71).

#### 4.7.1 Autophagy

Autophagy is a physiological process of cellular degradation and recycling in response to stress and damage (72). Autophagy is complex and can function both as a tumor suppressor by progressive cellular consumption or as a tumor promoter by sustaining survival through catabolic degradation (72). In other cancer types, metformin has been reported to both stimulate and inhibit autophagy (73). In this review of HNSCC, studies demonstrate that metformin can both decrease (74) and increase (62, 75), autophagy.

**Figure 7:**
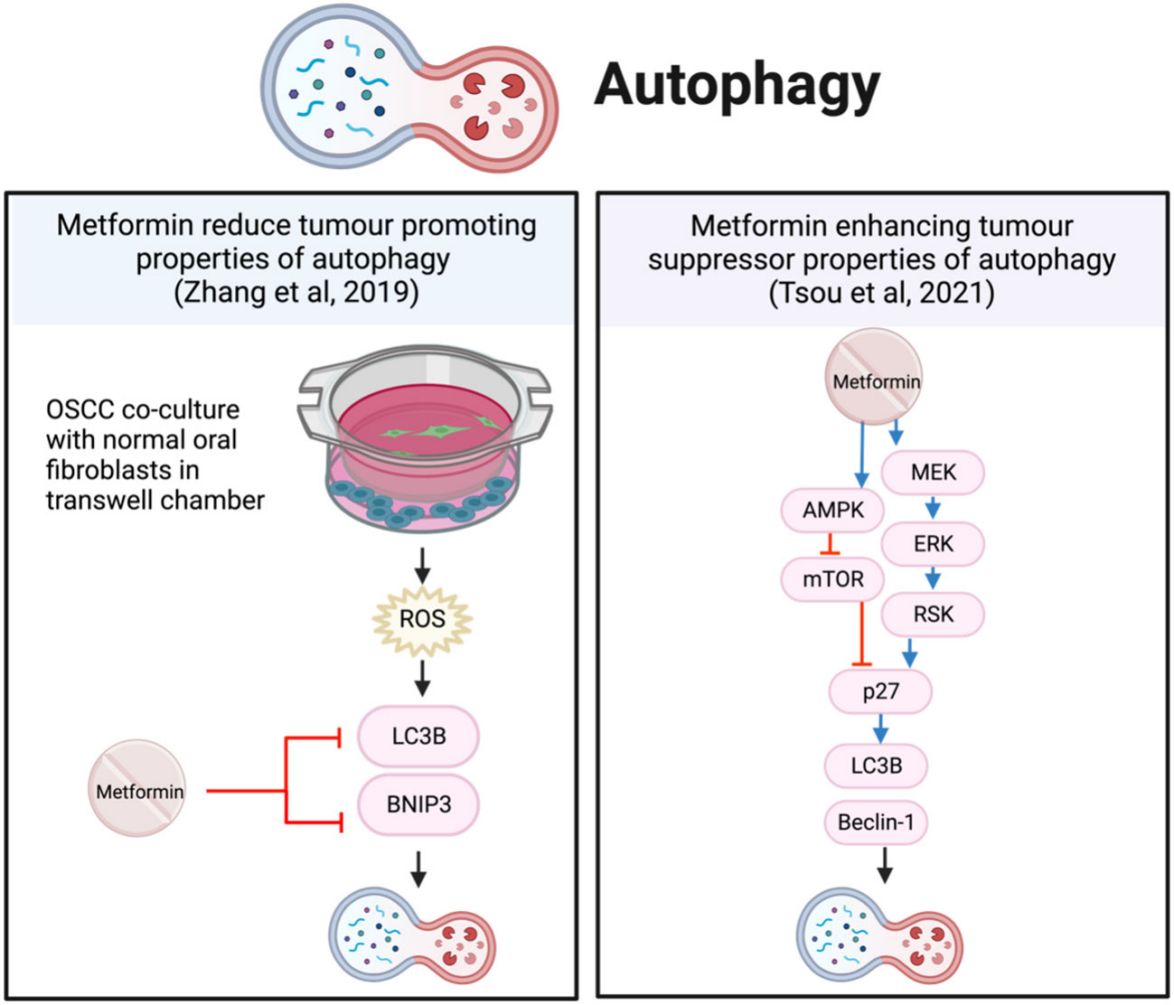
Metformin affects autophagy in HNSCC cells.

Metformin increased the markers of vesicle nucleation and elongation, LC3B and Beclin-1, which represented autophagy (62) (75). This involved activating p27 via both AMPK/mTOR and MEK/ERK/RSK pathways (62) (**Figure 7**). Even though metformin alone reduced cell viability and increased apoptosis and autophagy, the anti-tumor effects were further enhanced when metformin was combined with the autophagy inhibitor hydroxychloroquine *in vitro* and *in vivo* (75).

In contrast, primary OSCC cells co-cultured with normal oral fibroblasts (NOF) in transwell chambers, produced an environment with high concentrations of ROS and intercellular ATP, significantly increasing the growth of OSCC (74). The presence of NOFs promoted autophagy and mitophagy proteins, LC3B and BNIP3, and autophagosome-lysosome fusion to sustain proliferation. In this instance, metformin reduced the tumor-promoting autophagy by inhibiting LC3B and BNIP3 (74) (**Figure 7**). However, in this co-cultured OSCC model, NOFs inhibit AMPK by stabilizing the mitochondrial membrane potential and reduce metformin-induced apoptosis; thereby preventing a reduction in cell proliferation (74).

These studies present interesting and opposing effects of metformin on autophagy. The activation of autophagy by metformin in FaDu cells resulted in a dose-dependent reduction in cell viability (62). While metformin was reported to inhibit tumor-promoting autophagy initiated by NOFs in the co-culture model, it did not result in a reduction in OSCC cell proliferation (74). The NOFs represent the tumor microenvironment, better reflecting the *in vivo* interactions of cancer cells with their surrounding supporting cells. The protective effect of NOFs and other environmental factors may be overlooked in monocultures and warrants consideration in future research.

### 4.8 Reducing invasion and metastasis

**Figure 8:**
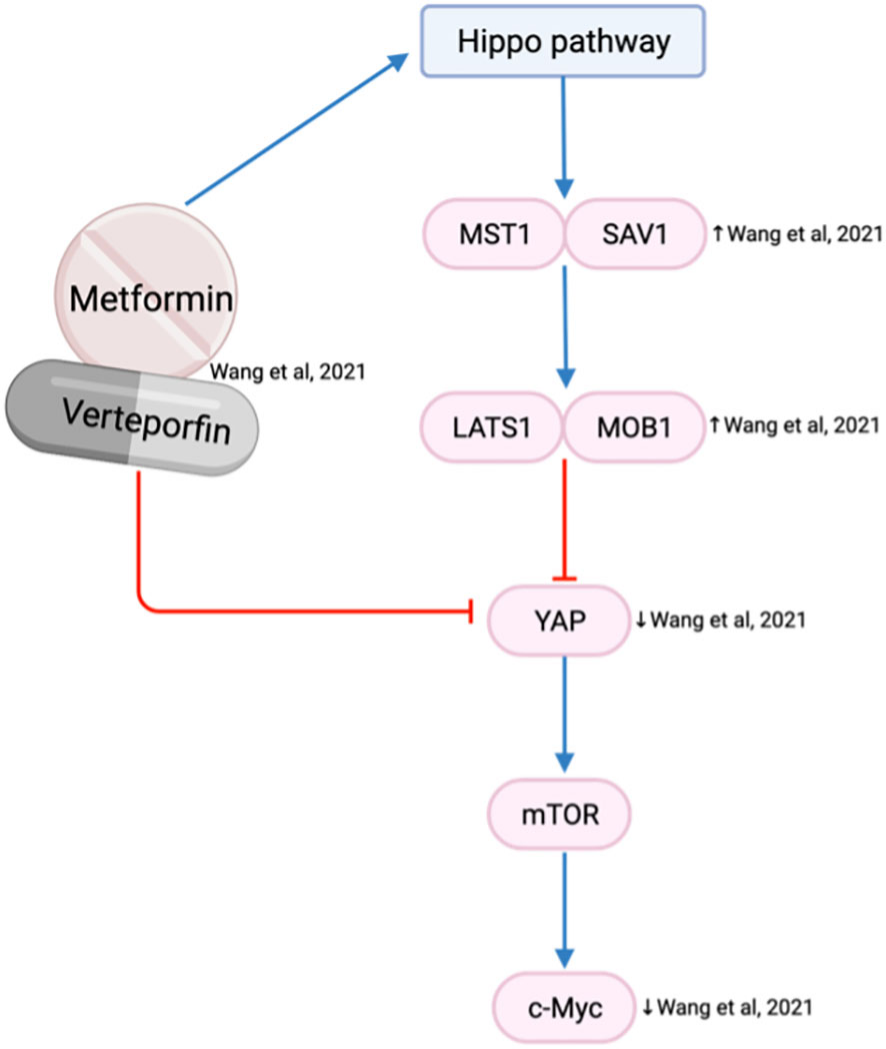
Metformin and verteporfin on the Hippo/YAP pathway.

The Hippo pathway plays a critical role in modulating cell proliferation and cellular phenotypes (76). Yes-associated protein (YAP) is an oncogene within the Hippo pathway that is frequently overexpressed in the HNSCC invasive front and associated with tumor aggressiveness, nodal metastasis, epithelial-mesenchymal transition (EMT) progression and drug resistance (76). Metformin is reported to activate the Hippo pathway, which reduces the expression of YAP and subsequent mTOR and c-Myc expression (63) (**Figure 8**). As a result, the combination of metformin and verteporfin, a YAP inhibitor, has an additional inhibitory effect on cell cycle and proliferation than either agent alone (63).

#### 4.8.1 Epithelial-mesenchymal transition

**Figure 9:**
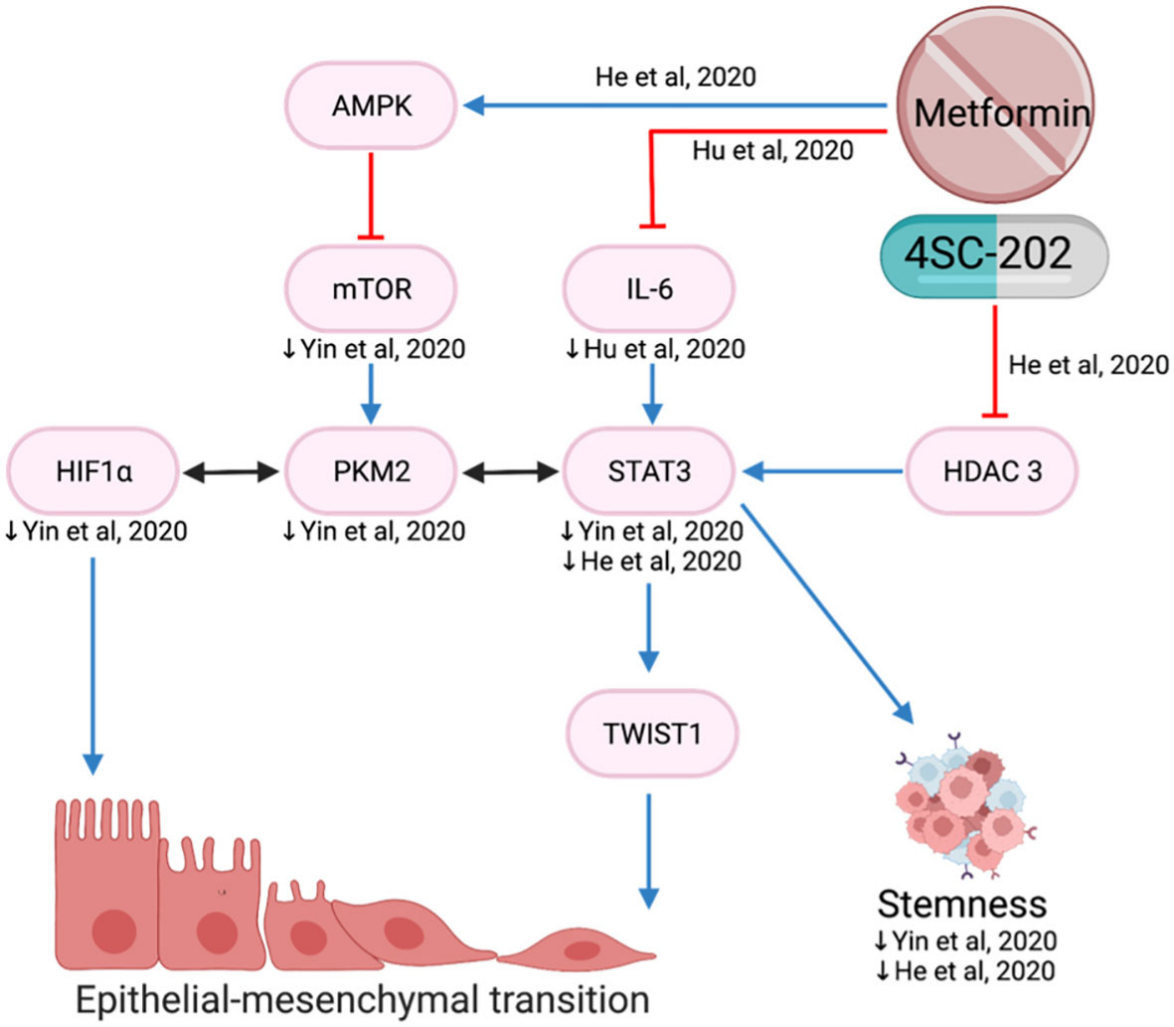
Metformin inhibits epithelial-mesenchymal transition.

EMT plays a key role in cancer progression, invasion, and metastasis. It is a biological process of polarized epithelial cells transitioning to mesenchymal phenotypes and is frequently initiated and regulated by hypoxia and the STAT3-TWIST pathway (77, 78). In the hypoxic environment, metformin reduced proliferation, migration, invasion and EMT by decreasing mTOR, HIF-1a, PKM2, STAT3 and EMT markers (78) (**Figure 9**).

Upregulation of TWIST1 promotes EMT and cancer invasiveness which is associated with poorer prognosis (79). Metformin and histone deacetylase inhibitor, 4SC-202, reduced EMT via the STAT3/TWIST1 axis (80). The drug combination is proposed to target STAT 3 via two different mechanisms: metformin suppresses STAT3 through activation of the AMPK/mTOR pathway and 4SC-202 suppresses histone deacetylase 3 enzymatic activity, preventing it from forming a complex to activate STAT3 (80) (**Figure 9**).

### 4.9 Nonmutational epigenetic reprogramming

Epigenetic regulation alters gene expression, without altering the DNA sequence, by DNA methylation, histone modification or through the effects of non-coding RNA (81).

#### 4.9.1 DNA Methylation

**Figure 10:**
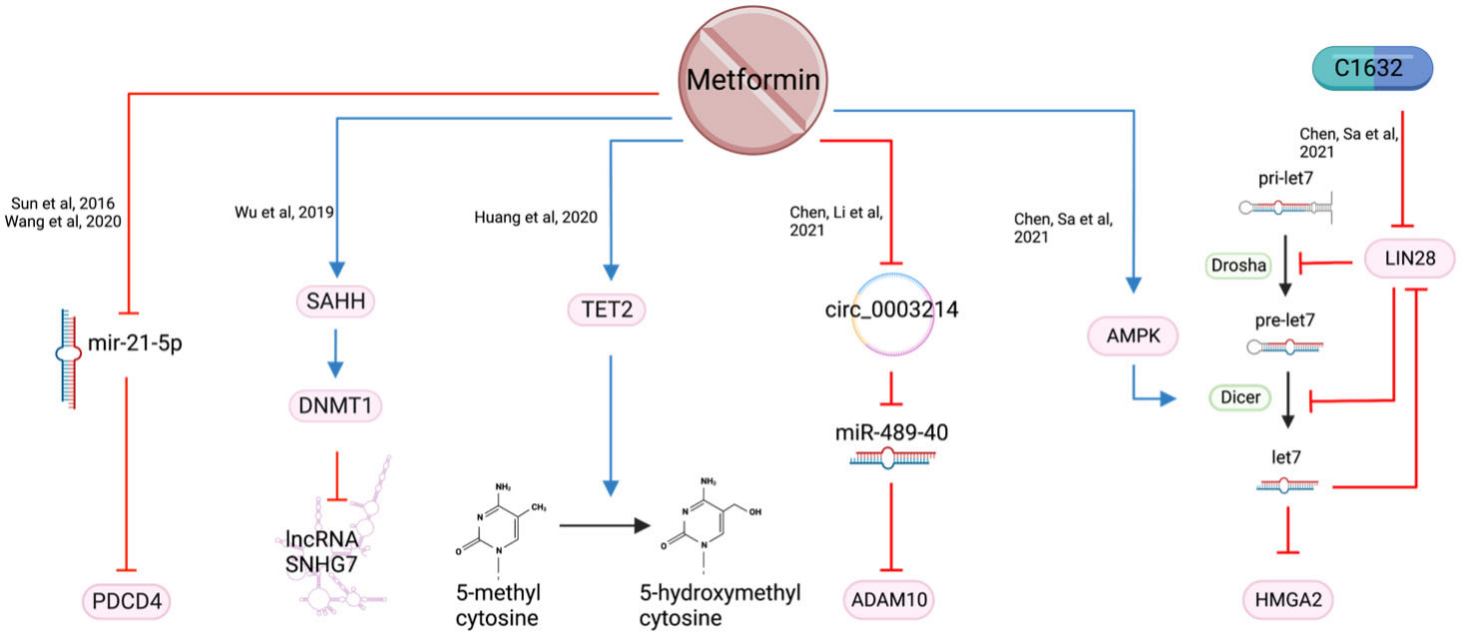
The epigenetic consequences of metformin exposure.

Tet methylcytosine dioxygenase 2 (TET2) is considered a tumor suppressor protein which converts 5-methylcytosine (5mC) to 5-hydroxymethylcytosine (5hmC) (82). Expression of TET2, and subsequently 5hmC, are reduced in HNSCC resulting in an alteration of gene expression to develop more aggressive features (83). Metformin, along with other treatments such as 5-azacytidine and Vitamin C are reported to increase the expression of TET2 and 5hmC (**Figure 10**), resulting in reduced proliferation and migration, but increased apoptosis (83).

#### 4.9.2 Non-coding RNA

Small nucleolar RNA host gene 7 (SNHG7) is a long non-coding RNA with oncogenic potential. It affects several transcription factors and signaling pathways by targeting microRNAs (miRNA) (84). High expression of SNHG7 correlates with increased tumor size, differentiation, advanced TNM staging, metastases, taxol chemotherapy resistance and radio resistance (85). Metformin was reported to inhibit HNSCC cell growth and increase apoptosis by silencing SNHG7 through the activation of S-adenosylhomocysteine (SAH) which upregulates DNA methyltransferase 1 (DNMT1), leading to increased methylation of the SNHG7 promoter (85) (**Figure 10**). Metformin’s action is likely a result of activating the S-adenosylhomocysteine hydrolase (SAHH), an enzyme that hydrates SAH into adenosine and homocysteine (86, 87). By targeting SNHG7, metformin appears to increase chemosensitivity to taxol and increase radiosensitivity (85).

MicroRNA-21-5p (miR-21-5p), a known oncogenic miRNA (88), is often over-expressed in HNSCC. It reduces the expression of the tumor suppressor gene Programmed Cell Death 4 (*PDCD4*) (89). Metformin, when used at supraphysiological doses of 50-100 mM, is reported to reduce miR-21-5p and increase the expression of PDCD4 (30, 90) (**Figure 9**). However, a metformin dose of 50-100 mM is unlikely to be achievable *in vivo* and therefore difficult to translate clinically.

LIN28 is a major developmental regulator and, therefore, an important target in cancer research. It can transform cancer cells into cancer stem cells (91) by inhibiting the maturation process of tumor suppressor miRNA let-7 (92). When metformin was combined with LIN28 inhibitor, C1632, it demonstrated a reduction in proliferation, migration, invasion *in vitro*, and a reduction in tumor weight and size *in vivo (* 91). In addition, metformin induces Dicer expression via AMPK (93), which is key to the maturation of pre-let7 to miRNA let7 (91). HMGA2 is a non-histone chromosomal protein that regulates chromatin structure which promotes cell cycle entry and inhibition of apoptosis in human malignancies (94). It is a downstream target of tumor suppressor let-7 and was synergistically reduced when OSCC cell lines were treated with the C1632 and metformin combination. The proposed mechanism of this synergistic outcome of metformin and C1632 is two-pronged - C1632 inhibits LIN28, allowing the primary mRNA (pri-let7) to be converted to let7; metformin activates AMPK which activates Dicer, converting pre-let7 to let7 (91) (**Figure 10**).

Metformin was also found to target circular RNAs (circRNA) which regulate miRNAs (95). Circ_0003214 was noted to be highly expressed while tumor suppressor miR-489-3p was under-expressed in HNSCC (95). Increased level of ADAM10 in OSCC is reported to contribute to migration and invasion (96). Metformin reduces circ_0003214 which subsequently increases miR-489-3p and reduces its target ADAM10 (95) (**Figure 10**). This was reflected in the reduction of cell viability and colony formation *in vitro* and reduced tumor weight and volume *in vivo* (95).

#### 4.9.3 Alternative splicing

Aberrant splicing events can result in cancer (97). Metformin is reported to be involved in 1521 alternative splicing events involved in processes including: centrosome function, DNA damage, ATPase activity, RIG-I-like receptor signaling pathway and Wnt signaling (98). Among them, exon 3 skipping of nucleotide binding protein 2 (NUBP2), an ATP-binding protein, was increased following metformin treatment (98). Metformin induces a splicing switch to the canonical long isoform, which in turn reduces intracellular ATP to inhibit cancer cell proliferation and reduce colony formation (98).

#### 4.9.4 Histone modification

Metformin treatment of HNSCC cell lines induced acetylation of the lysine residue of histone H3 protein (H3K27ac) (32). H3K27ac is an enhancer of transcription. Transcriptomic profiling revealed metformin affects transcripts involved in the cell cycle, ribosome, oncogenes, tumor suppressors, metabolism and cytokines via H3K27ac (32).

### 4.10 Cancer stem cells

Cancer stem cells (CSCs) are a small subpopulation of the cells within a tumor that have self-renewal and differentiating abilities. These cells are easily cultured and highly tumorigenic with distinct surface markers such as CD44, CD133 and ALDH (99).

**Figure 11:**
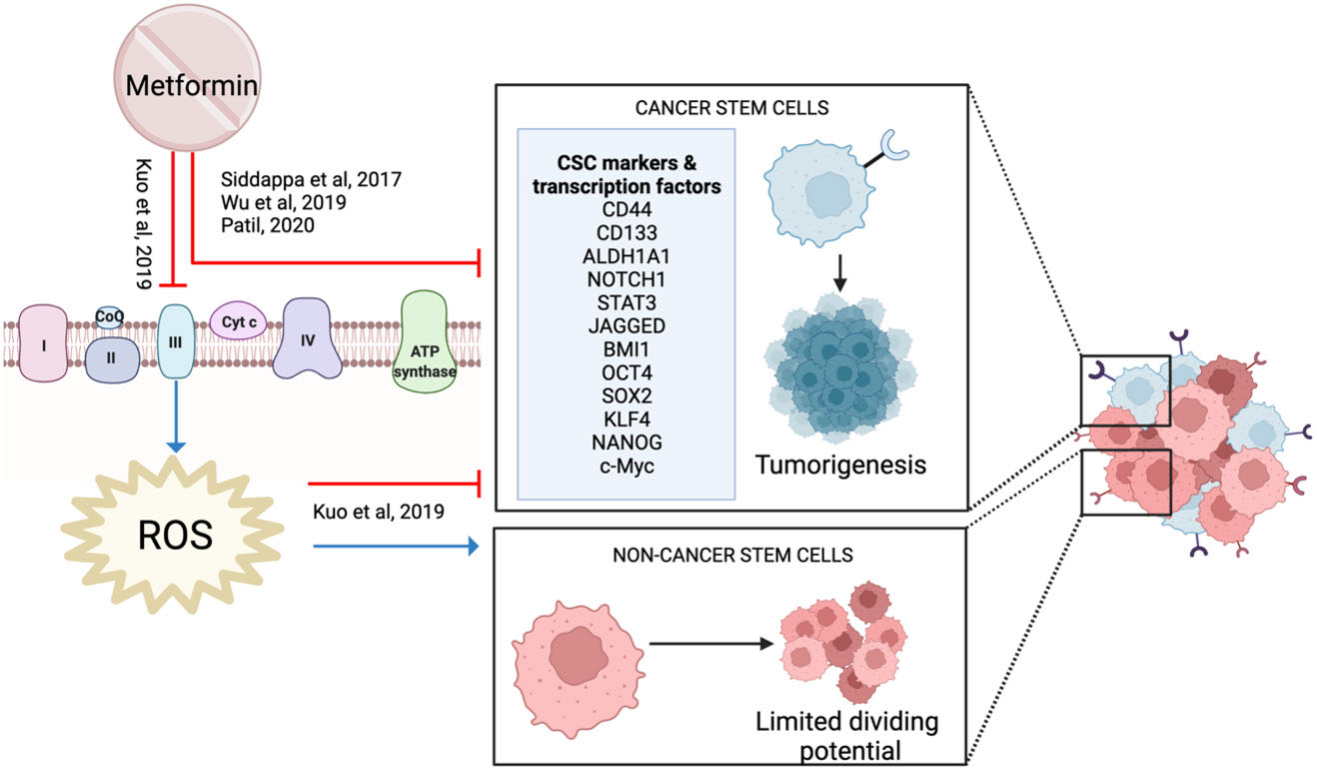
Metformin and cancer stem cells.

Metformin reduced HNSCC CSC markers (NOTCH1, STAT3, CD44, JAGGED) and stemness-related transcription factors (OCT4, SOX2, KLF4, c-Myc, NANOG), suggesting it has an impact on CSCs (29, 56, 100) (**Figure 11**). When combined with a high dose of curcumin (100 μM), it reduced proliferation and increased apoptosis in cancer cell lines (53). This activity was supported in a 4NQO rodent model when metformin was combined with low-dose curcumin, demonstrating decreased carcinogenesis progression with reduced expression of NF-kB, pS6 and the CSC profile (100).

In contrast, Kuo et al. showed that low-dose metformin increased cell proliferation in laryngeal cancer stem cells and ALDH+ CSC, but reduced proliferation in ALDH- non-CSC (101). Metformin increased stemness markers and protected CSCs against cisplatin. However, this protective effect was not observed in non-stem cells, resulting in a reduction in proliferation (101). Metformin is known to inhibit mitochondrial complex I. However, *in vitro* and *in silico* data suggest that metformin binds strongly and inhibits mitochondrial complex III activity, which reduces ROS production (101). Reduced ROS is essential to maintain and promote CSC, whereas it has the opposite effect on non-CSC (101) (**Figure 11**). Therefore, Kuo et al. proposed that metformin reduces proliferation by targeting non-CSC, but protected CSCs which then causes treatment resistance. This study is important to highlight the heterogeneity of a cancer cell population and how the different cell types within the tumor may respond differently to metformin.

Patient-derived cancer organoids are generated from pluripotent stem cells and differentiate into a three-dimensional complex structure of various cell types (102). This model, consisting of CSCs, allows a more accurate representation of human cancer and may affect metformin’s anti-proliferative activity.

### 4.11 Tumor microenvironment

Tumors are surrounded by fibroblasts, immune cells, inflammatory cells, epithelial, endothelial, mesenchymal cells, and the extracellular matrix, Together, they make up the tumor microenvironment (103). The tumor microenvironment plays an active role in the promotion of tumor survival and progression. This section discusses how metformin can affect various factors in the tumor microenvironment.

#### 4.11.1 Stromal fibroblasts

Cancer-associated fibroblasts (CAFs) surround the tumor and secrete various factors to support and promote cancer cell growth (104). Co-culturing primary OSCC with normal oral fibroblasts (NOF) increases proliferation and autophagy (74) as discussed earlier (Section 5.7.1).

**Figure 12:**
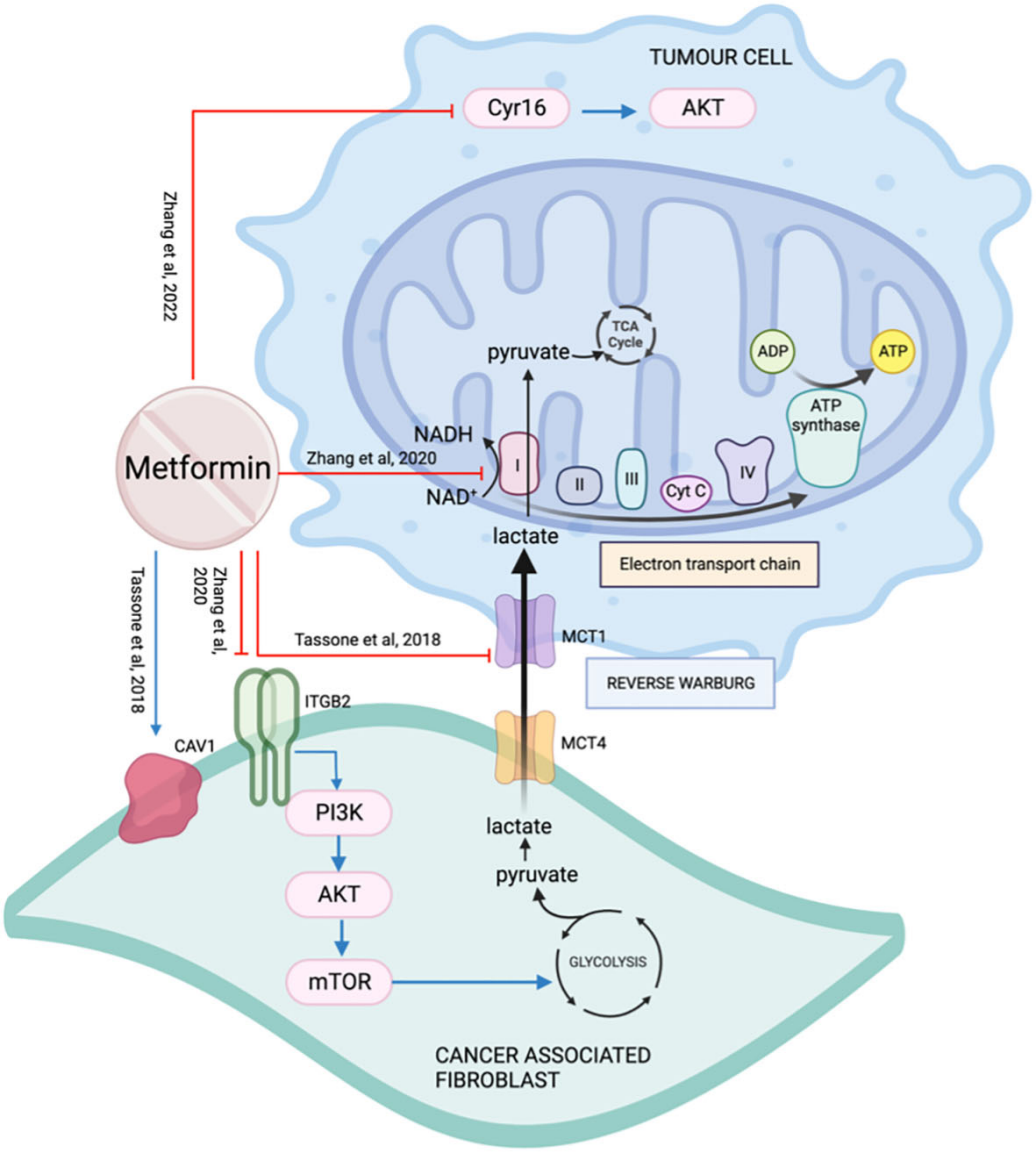
The effect of metformin on the tumor microenvironment.

Metabolic coupling, also termed “reverse Warburg effect”, occurs when the cancer stroma metabolically supports the cancer cells by catabolite transfer. Monocarboxylate transporters (MCTs) are essential for the cancer cells to undergo metabolic coupling. Caveolin-1(CAV1) is a membrane protein frequently downregulated in CAFs and associated with tumor aggressiveness (105). Xenografts with CAV1 knockdown fibroblasts did not show a significant impact on tumor growth when treated with metformin, suggesting CAV1 is essential for metformin’s action (105). In a co-injection rodent model with fibroblasts, treatment with metformin decreased metabolic coupling by reducing cancer cell MCT1 and restoring fibroblast CAV1 expression (105) (**Figure 12**).

Integrin beta 2 (ITGB2) is a subunit of integrin and a cell surface glycoprotein that regulates cell survival, proliferation, and movement. It is involved in the control of metabolic pathways by coordinating intracellular and extracellular signaling (106). ITGB2 is upregulated on OSCC CAFs membrane and cytoplasm. It is associated with higher TNM staging, greater depth of invasion, shorter survival, and early recurrence (106). The presence of ITGB2, whether on CAFs or overexpressed on NOFs, was able to promote tumor cell proliferation by activating the PI3K/AKT/mTOR pathway resulting in increased glycolysis, generating pyruvate which is then converted to lactate. Lactate exits CAFs through MCT4, enters cancer cells through MCT1, and is used by mitochondrial complex I to generate NADH (106) (**Figure 12**). Metformin targets the electron transport chain, disrupting the availability of NAD+ that is required for the oxidation of lactate for cancer cells. Zhang et al. concluded that even though the presence of ITGB2 on the CAFs in the tumor microenvironment promotes OSCC proliferation, it also makes them more sensitive to the effect of metformin.

Cellular communication network factor 1 (CCN1/Cyr16) is a regulatory protein of the tumor microenvironment and is over-expressed in many cancers including HNSCC (107). Metformin reduces Cyr16 and its downstream target p-AKT resulting in a reduction in cell viability in cell line SCC25 (108) (**Figure 12**).

#### 4.11.2 ΔNp63

**Figure 13:**
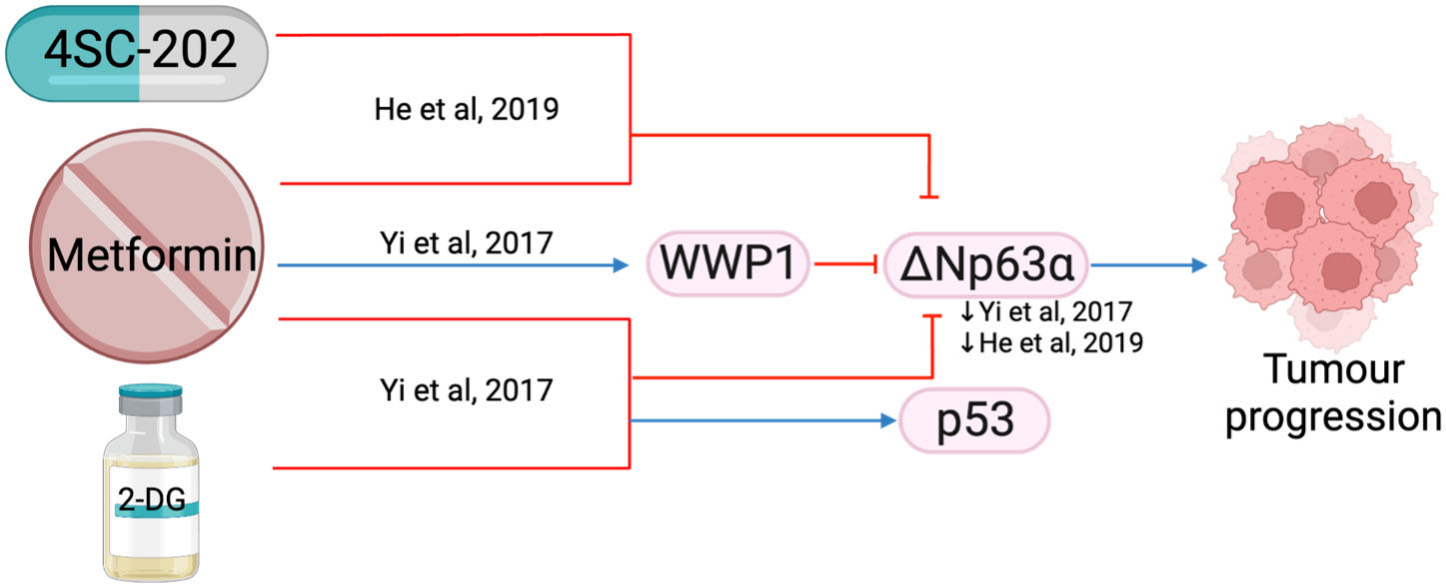
The effect of metformin in combination with other treatments (4SC-202 and 2-DG) on ΔNp63a.

ΔNp63 is the predominant p63 isoform and is frequently overexpressed in HNSCC (109). It is essential for regulating adhesion molecules and can influence the tumor microenvironment (110). The combination of metformin with histone deacetylase inhibitor, 4SC-202, synergistically reduced cell proliferation and colony formation *in vitro*, and reduced tumor weight and volume *in vivo (* 71). The combination treatment destabilized oncogene ΔNp63 in a post-translational manner by increasing protein ubiquitination and proteasome-mediated degradation through ubiquitin E3 ligase, WWP1(**Figure 13**) (111), which led to reduced fibronectin resulting in reduced cell matrix adhesion (111) and increased apoptosis (71). Metformin in a glucose-deprived environment, or used in combination with 2-DG, further enhanced the effects of reduced ΔNp63 (**Figure 13**) (111).

#### 4.11.3 Nerve growth factor receptor

**Figure 14:**
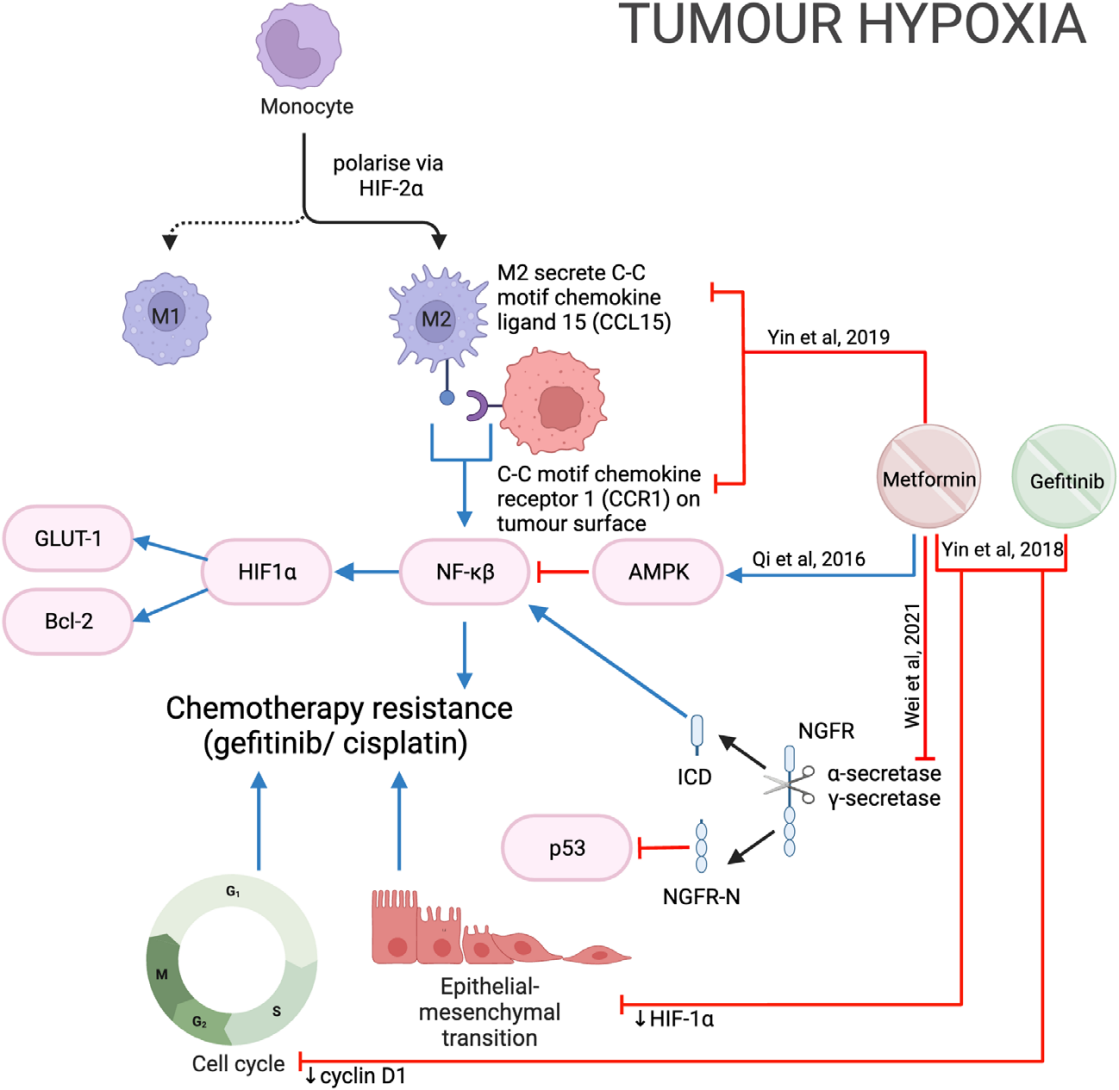
Metformin in the hypoxic tumor microenvironment.

Nerve growth factor receptor (NGFR) is a transmembrane protein that promotes cell survival, and its overexpression in OSCC is linked with poorer prognosis (112). Proteolytic processing withɑ-secretase and 𝛾-secretase cleaves NGFR into two components: NGFR-N and the intracellular domain (ICD). NGFR-N degrades p53 (113), while ICD activates the NF-𝜅B pathway (114), both leading to tumor progression. Metformin was shown to inhibit NGFR proteolysis by reducing the expression of ɑ-secretase and 𝛾-secretase genes, thereby reducing cell proliferation and growth (**Figure 14**).

#### 4.11.4 Tumor hypoxia

The rapid proliferation of cells and lack of vasculature within a solid tumor can result in tumor hypoxia, which contributes to chemo- and radio-resistance (115). Higher cisplatin concentrations were required when HNSCC cells were grown in a hypoxic (1% O_2_) compared to a normoxic (20% O_2_) environment (116). Metformin was shown to inhibit hypoxia-induced NF-𝜅B and thereby downregulate HIF-1a expression and downstream proteins GLUT-1 and Bcl-2 *in vitro* and *in vivo*, potentially through the activation of AMPK (116) (**Figure 14**). Qi et al. then showed the IC_50_ of cisplatin was significantly less when used in combination with metformin during hypoxia, suggesting metformin sensitizes HNSCC cells to cisplatin and reduces chemoresistance despite hypoxia (116). However, it is important to also note that metformin has been reported to protect esophageal SCC from cisplatin by reducing the formation of cisplatin-DNA adduct complexes (117). These two studies (116, 117) differ in the concentration of oxygen and may suggest metformin is more effective against cells in a more hypoxic environment.

Gefitinib is an effective epidermal growth factor receptor tyrosine kinase inhibitor in lung cancer but has not been approved by the FDA for use in HNSCC (118). Hypoxia has also been shown to induce gefitinib resistance by inducing EMT (118). In HNSCC *in vitro* and *in vivo* models the combination of metformin and gefitinib reduced HIF-1ɑ, cyclin D1 and EMT to overcome hypoxia-induced gefitinib resistance (118) (**Figure 14**). Clinical trials combining metformin and gefitinib are underway in lung cancer (NCT01864681, NCT03071705; www.ClinicalTrials.gov), however, such trials have not been considered for HNSCC. These two studies (116, 118) suggest that the addition of metformin to cisplatin and gefitinib may reduce the development of hypoxia-induced chemoresistance in HNSCC and may be worth further investigation.

Tumor hypoxia contributes to radio resistance, and metformin was reported to increase tumor oxygen saturation and hemoglobin concentration using non-invasive photoacoustic imaging in an orthotopic xenograft rodent model (119). This study suggests that metformin may target tumor tissue and supports its use as a radiosensitizer (119). It is the first study that demonstrated metformin modulates tumor oxygenation in HNSCC without increasing the vessel count. This suggests metformin increased tumor oxygen saturation by reducing oxygen consumption (119). However, the benefit of metformin is temporary, with oxygen saturation returning to baseline 48 hours after cessation.

#### 4.11.5 Immune modulation

The immune microenvironment plays a role in protecting tumors from chemotherapy by producing pro-tumor mediators (120). Tumor-associated macrophages (TAM) contribute to chemotherapy resistance (121). Hypoxia in the tumor microenvironment polarizes macrophages to M2-macrophages (M2-TAM) via HIF-2ɑ, which secretes C-C motif chemokine ligand 15 (CCL15). CCL15 then reacts with the C-C motif chemokine receptor (CCR1) on the tumor cell surface to activate NF-𝜅B, promoting gefitinib resistance. Metformin was found to inhibit CCL15 secretion by M2-TAM and reduce CCR1 expression in HNSCC cells (**Figure 14**); therefore, reducing the development of drug resistance when used in combination with gefitinib (122).

Metformin has also been reported to impact both the peripheral blood and tumor infiltrating natural killer (NK) cells (123). Metformin-treated NK cells increased NK cell cytotoxicity compared to vehicle control. This is likely a result of metformin increasing pSTAT1, which stimulates an increase in perforin secretion. Perforin plays an important role in forming transmembrane pores and subsequent apoptosis in tumor cells (124). Metformin also inhibits mTOR and pSTAT3, with subsequent impact on the tumor promoting chemokine C-X-C motif ligand 1 (CXCL1). This prevents interaction with CXC receptor 2, which then reduces the proliferation and progression of cancer cells (125). A comprehensive diagram has been included in Crist et al’s paper and therefore not replicated here (123).

### 4.12 Polymorphic microbiomes

The tumorigenesis of HPV-positive HNSCC relies on the inhibition of p53 by HPV viral proteins E6 and E7. Hoppe-Seyler demonstrated that metformin was able to reduce E6 and E7 expression in HPV-positive HNSCC and cervical cancer cells in a dose- and time-dependent manner, but this was reversible after changing to metformin-free media (126). Exploration of the mechanistic pathways has only been performed in cervical cancer cell lines HeLa and SiHa cells and not in HNSCC, but the results suggested that PI3K is key in the suppression of E6 and E7 proteins. Metformin induced only a reversible reduction in the oncoproteins, suggesting senescence was not achieved. Furthermore, metformin also counteracted the pro-senescent effects when combined with chemotherapeutic agents Etoposide and Doxorubicin in this study (126).These findings suggest that metformin could potentially interfere with senescence in the presence of oncoproteins E6 and E7 and should be used with caution when combined with other chemotherapy drugs. This should also be further investigated before extrapolating the results to HPV-positive HNSCC cell lines.

### 4.13 Limitations of the included studies

Several limitations need to be acknowledged when translating these pre-clinical findings into clinical treatment. The doses of metformin used for *in vitro* studies are generally much higher in concentration than the reported physiological concentrations in the systemic circulation, with one *in vitro* study using up to 100 mM metformin (30). Therapeutic doses of metformin in human systemic circulation, approximately 10-40 μM, may not be sufficient to affect tumor growth (127), therefore alternative modes of delivering metformin to the tumor cells may need to be explored. Song et al. designed a metformin-cisplatin nanoparticle that is activated by laser and targets ligand low-density lipoprotein receptor which is specific to the hypoxic centers of HNSCC (128). Metformin sensitized the effect of cisplatin in cell culture and *in vivo*. This novel method of drug delivery was able to target the xenograft tumor with high doses of cisplatin (10 mg/kg) combined with metformin (1 mg/kg) to overcome the low systemic concentration of metformin and reduce potential toxicities. Drug delivery methods incorporating metformin in micro- and nanoparticles are currently being explored to overcome low oral bioavailability and to target dose delivery for effective cancer treatment (129).

Cancer cell culture provides an invaluable model for investigating mechanistic pathways, but several factors may limit the relevance to responses in human disease. Cell culture growth medium provides a protected environment favoring cell growth, containing high concentrations of glucose, essential nutrients, growth factors, various hormones, and antibiotics, which are non-physiological. As a result, cells growing in this favorable environment may require higher doses of metformin. The *in vitro* studies discussed largely use cancer cells grown in a monolayer. Again, this is not reflective of the *in vivo* environment where solid tumors are 3D structures, consisting of differentiated cell types and CSC, constantly influenced by their microenvironment. This review discussed the different impact metformin has on CSC (101), hypoxic regions (116) and surrounding cells (105). 3D models such as spheroids or organoids can potentially address some of these issues.

The *in vivo* animal studies discussed used varying doses of metformin, with diverse methods of administration (oral intake, intraperitoneal injection or xenografts pre-treated with metformin), for a variable duration of treatment. Furthermore, xenograft models largely employ immunodeficient animals, which do not fully reflect how the human immune system interacts with cancer development. None of the studies measured metformin concentration in the animal systemic circulation, nor correlated the doses to the human systemic circulation, which is worth exploring.

In most of these pre-clinical studies, HNSCC cells have been classified as a single entity, whereas clinically HNSCC vary significantly depending on the subsite origin, differentiation status, and HPV status (currently only clinically relevant to oropharyngeal SCC). These factors impact pathophysiology, disease development and treatment response. Moving forward, a better description of cell line characteristics (including subsites, HPV status, and mutations) will be important for accurate interpretation of metformin response, characterization of the molecular pathways involved, and clinical translation.

### 4.14 Summary

This scoping review provides a comprehensive presentation of the biological actions of metformin in HNSCC in the pre-clinical setting. A vast array of pathways has been presented and discussed, with many impacting the hallmarks of cancer. While the AMPK/mTOR pathway is the most well-described mechanistic action of metformin in HNSCC, there is a growing focus on metformin’s role in epigenetic regulation and the surrounding tumor microenvironment. It is important to be aware that even though each study focuses on a select pathway, metformin acts through many pathways simultaneously.

A recently updated meta-analysis, including 14694 patients in 11 cohort studies, concluded that adjuvant metformin use benefits overall survival, disease-free survival and disease-specific survival in HNSCC patients (130). This review raises the concept that HNSCC disease stratification, such as OCT3 expression, *TP53* mutation, and HPV status, may improve the clinical outcomes when using metformin. The response to metformin may also be affected by the surrounding tumor microenvironment, hypoxia and the heterogeneity within each tumor.

These studies are encouraging and support ongoing research into the use of metformin as an adjuvant treatment for HNSCC. However, epidemiological studies suggest that metformin is unlikely to be used as a monotherapy to effectively treat HNSCC. Combining metformin with existing or novel treatments provides an opportunity to exploit the benefit of metformin at lower doses. This review highlights additive or synergistic responses when metformin is combined with other therapies, ranging from compounds such as curcumin to radiation treatment. Validation in 3D models using co-cultured spheroids or organoids could provide valuable and novel information on metformin’s impact on surrounding cells, hypoxia and cancer stem cells.

## 5 Conclusion

Metformin is a widely used medication with a proven safety profile that demonstrates anti-tumor properties in HNSCC. This comprehensive scoping review of the pre-clinical literature summarizes the proposed mechanisms of action of metformin on HNSCC cancer cells, the surrounding tumor microenvironment, and animal models. Further validation of metformin combinations in 3D cancer models will provide interesting and valuable information. It is important to consider the limitations of *in vitro* and *in vivo* animal models when designing clinical trials to further explore the benefit of metformin. Metformin is at the forefront of drug repurposing and presents an exciting and promising agent for use as an adjuvant therapy in the treatment of HNSCC.

## Data availability statement

The original contributions presented in the study are included in the article/**Supplementary Material**. Further inquiries can be directed to the corresponding author.

## Author contributions

LH: Conceptualization, Data curation, Funding acquisition, Methodology, Writing – original draft, Writing – review & editing. CW: Supervision, Validation, Writing – review & editing. ND: Data curation, Methodology, Writing – review & editing. MM: Supervision, Writing – review & editing. EO: Supervision, Writing – review & editing.
