# Supplementary material for: The mechanisms of action of metformin on head and neck cancer in the pre-clinical setting: a scoping review
Source: Front Oncol. 2024 Feb 22;14:1358854. doi: 10.3389/fonc.2024.1358854 (PMC10917904; doi:10.3389/fonc.2024.1358854)
Supplement: Supplementary file 1 [file DataSheet_1.pdf]

Supplementary Material

The mechanisms of action of metformin on head and neck cancer in the pre-clinical setting: a scoping review

Lucy Huang*[1,2], Charmaine M Woods[1,2], Nuwan Dharmawardana[1,2], Michael Z Michael[1,3], Eng Hooi Ooi[1,2]

*** * Correspondence:**
Lucy Huang
lucy.huang.tw@gmail.com

1 Supplementary Data

Supplementary Material should be uploaded separately on submission. Please include any supplementary data, figures and/or tables.

Supplementary material is not typeset so please ensure that all information is clearly presented, the appropriate caption is included in the file and not in the manuscript, and that the style conforms to the rest of the article.

2 Supplementary Figures and Tables

For more information on Supplementary Material and for details on the different file types accepted, please see here.

2.1 Supplementary Figures

Supplementary Figure 1. The figure legends are required to have the same font as the main text, 12 point normal Times New Roman, single spaced. Please use a single paragraph for each legend and prepare the figures keeping in mind the PDF layout.

Supplementary Table 1. Search terms and keywords

#	Searches
1	exp "head and neck neoplasms"/ or "squamous cell carcinoma of head and neck"/ or exp mouth neoplasms/ or exp palatal neoplasms/ or exp tongue neoplasms/ or exp otorhinolaryngologic neoplasms/ or exp laryngeal neoplasms/ or pharyngeal neoplasms/ or hypopharyngeal neoplasms/ or oropharyngeal neoplasms/ or tracheal neoplasms/ or tonsillar neoplasms/
2	((cancer* or carcinoma or SCC or tumour* or tumor* or malignan* or neoplasm* or oncolog*) adj4 (oral or tongue or buccal or oropharynx or oropharyn* or laryn* or pharyn* or hypopharyn or glotti* or supraglotti* or vocal or palat* or head or neck or tonsil* or HN or superglot* or OPSCC or "OP-SCC" or SCCHN or HNSCC or HNC)).tw,kf.
3	or/1-2
4	Metformin/
5	(metformin or "1,1-dimethylbiguanide" or dimethylguanylguanidine or dimethylbiguanidine or glucophage* or 9100l32l2n or 786z46389e).tw,kw.
6	or/4-5
7	3 and 6

Supplementary Data 1. Description of the included studies.

44 studies conducted *in vitro* work with cell lines, 25 conducted *in vivo* studies in 4NQO induced or xenograft mice models. Metformin was also combined with other drugs including curcumin(1, 2), adriamycin(3), 2-deoxyglucose (2-DG)(4-6), cisplatin(7-9), C1632(10), radiation(11-13), 4SC-202(14, 15), dichloroacetate(16), LY2835219 ((17), 5-fluorouracil(18), gefitinib(19-21), dasatinib(22), verteporfin(23), taxol(12) and bis-2-ethyl sulfide (BPTES)(24). Despite a search with no time limit, pre-clinical studies of metformin use in HNSCC were only published from 2011 onwards.

Supplementary Table 2. Study characteristics of the included studies.

Characteristics		Number
Year Published	2011	1
	2012	6
	2013	1
	2014	1
	2015	2
	2016	5
	2017	4
	2018	3
	2019	10
	2020	7
	2021	7
	2022	4
	2023 (published before May)	1

Country published	China	26
	USA	15
	Taiwan	3
	Canada	2
	Japan	2
	Saudi Arabia	1
	Turkey	1
	Brazil	1
	India	1
Types of studies	In vitro: metformin alone	44
	In vitro: metformin + additional treatment	24
	In vivo: metformin +/- additional treatment	25

Supplementary Table 3. Studies that investigate the effect of metformin *in vitro* and/or *in vivo*

Year	In vitro: metformin alone	In vitro: metformin + additional treatment	In vivo: metformin +/- additional treatment	Reference
2011		Sandulache et al		(25)
2012	Luo et al		Luo et al	(26)
	Ma et al	Ma et al		(21)
	Sandulache et al	Sandulache et al		(5)
	Sikka et al			(27)
	Vitale-Cross et al		Vitale-Cross et al	(28)
		Skinner et al	Skinner et al	(13)
2013	Patel et al			(29)
2014	Lin et al	Lin et al	Lin et al	(30)
2015	Madera et al		Madera et al	(31)
	Sun et al			(32)
2016	Guimaraes et al			(33)
	Harada	Harada	Harada et al	(18)
	Qi et al	Qi et al	Qi et al	(9)
	Siddappa et al	Siddappa et al	Siddappa et al	(2)
	Woo et al			(11)
2017			Thompson et al	(34)
	Chen et al		Chen et al	(35)
	Chen et al			(36)
	Yi et al	Yi et al	Yi et al	(6)
2018		Yin et al	Yin et al	(20)
			Verma et al	(37)
			Tassone et al	(38)
2019		Yin et al	Yin et al	(19)
	He et al	He et al	He et al	(14)
	Inanc et al	Inanc et al		(39)
	Lindsay et al	Lindsay et al		(1)
	Kuo et al	Kuo et al		(8)
	Wu et al		Wu et al	(40)
	Wu, Tang et al	Wu, Tang et al	Wu, Tang et al	(12)
	Yang et al	Yang et al		(24)
	Zhang	Zhang		(3)

	Zhang et al			(41)
2020		Song et al	Song et al	(7)
	He et al	He et al		(15)
	Hu et al	Hu et al	Hu et al	(17)
	Huang et al			(42)
	Patil et al			(43)
	Wang et al			(44)
	Zhang et al			(45)
2021	Chen et al	Chen et al	Chen et al	(10)
	Hoppe-Seyler et al			(46)
	Tsou et al			(47)
	Wang et al	Wang et al		(23)
	Wei et al			(48)
	Yin et al		Yin et al	(49)
	Chen et al		Chen et al	(50)
2022	Crist et al			(51)
	Liu et al		Liu et al	(52)
	Zhang et al			(53)
	Zhao et al	Zhao et al	Zhao et al	(54)
2023	Ji et al			(55)

Supplementary Table 4. Details of the cell lines in the included studies.

Cell line	Anatomical subsite	HPV status	Mutations
CAL27	Tongue	-	TERT, TP53
CAL33	Tongue	-	TMPRSS2, TP53, PIK3CA
Detroit 562	Oropharynx	-	TP53, aberrant SMAD4, overexpress EGFR and cyclin D1
Detroit 551	Skin (normal control)	-	N/A
FaDu	Hypopharynx	-	CDKN2A, FAT1, TP53, aberrant SMAD4, overexpress EGFR and cyclin D1
HaCat	Skin (normal control)	-	TP53
HGF1	Normal oral gingiva	-	N/A
HN4	Larynx	-	None documented
HN6	Tongue	-	None documented
HN12	Tongue LN derivative	-	None documented
HN13	Tongue	-	TP53
HN30	Oropharynx	-	None documented
HN30 ALDH+	Oropharynx	-	None documented
HN30 ALDH-	Oropharynx	-	None documented
HN31	Oropharyngeal (HN30) LN derivative	-	TP53

HSC2	Oral cavity LN derivative	-	PIK3CA, TP53
HSC3	Tongue LN derivative	-	CDKN2A, PIK3CA, TERT, TP53
HSC4	Tongue LN derivative	-	CDKN2A, PIK3CA, TERT, TP53
HSC6	Oral cavity	-	HRAS
JLO-1 CSC	Larynx	-	None documented
SAS	Tongue	-	TP53
SCC3	Nasal cavity and paranasal sinuses	-	TP53
SCC4	Tongue	-	TP53
SCC6	Tongue	-	CDKN2A, TP53
SCC9	Tongue	-	TP53
SCC15	Tongue	-	None documented
SCC25	Tongue	-	TP53
SCC31	Tongue	-	TP53
SCC90	Base of tongue	+	None documented
SCC131	Floor of mouth	-	None documented
SCC152	Hypopharynx	+	Not documented
UDSCC2	Hypopharynx	+	KMT2D
UMSCC47	Tongue	+	NOTCH1, TP53

The details of the mutations are taken from the cell-line reference resource Cellosaurus (https://web.expasy.org/cellosaurus/)(56)

Supplementary Table 5. Metformin in HNSCC: *in vitro* studies

Author	Year	Cell line	Metformin dose + duration	Metformin outcome	Mechanism of action
**Luo et al (26)**	2012	CAL27	5, 10, 20 mM	↓proliferation, ↓colony formation, ↑apoptosis (20mM)	G0/G1 arrest, apoptosis, ↑ p-AMPK, ↓ p-mTOR,↓ p-S6K, ↓cyclin D1, ↓p-pRb, ↓CDK4, ↓CDK6, ↓Bcl-2, ↓Bcl-xL, ↑Bax
		HN6			
		SCC25			
**Ma et al (21)**	2012	SCC9	1-10 mM for 24 hours	N/A	↑ADP/ATP ratio, ↑p-LKB1, ↑p-AMPK
		SCC25			
**Sandulache et al (5)**	2012	HN30	10-30 mM	↓colonies	↓oxygen consumption rates, ↑lactic acid production, demonstrating reduction of mitochondrial respiration
**Sikka et al (27)**	2012	FaDu	5-20 mM for 72 hours	↓total cell number, ↔cell death	↑AMPK → ↓4E-BP1 & ↓ EF2 → ↓protein expression of cyclins, Cdks, CDKI (↓cyclin D1, ↓cyclin E, ↓Cdk4, ↓Cdk6, ↓INK4B/p15, ↓INK4A/p16, ↓INK4C/p18, ↔INK4D/p19, ↓Cipl/p21, ↓Kipl/p27) → Cell cycle arrest (↑G1 phase, ↓S phase)
		Detroit 562		↓total cell number, ↔cell death	↑AMPK → ↓4E-BP1 & ↓EF2→ ↓protein expression of cyclins, Cdks, CDKI (↓cyclin D1, ↓cyclin E, ↓Cdk2, ↓ Cdk4, ↓Cdk6, ↓INK4B/p15, ↓INK4A/p16, ↓INK4C/p18, ↔INK4D/p19, ↔Cipl/p21, ↓Kipl/p27) → Cell cycle arrest (↑G1 phase, ↓G2/M phase)
	2012	HaCat	10-20 mM	↔cell proliferation	

**Vitale-Cross et al (28)**		Cal27		↓cell proliferation	↑p-AMPK, ↑p-ACC, ↓pS6 (in both LKB1 knockdown and control). Metformin inactivates mTOR, likely independent of AMPK
		HN12			
		HN13			
**Patel et al (29)**	2013	HN4	3 mM metformin for 3 days	No OCT3 expression - no change in viability	OCT3 is required for metformin uptake in HNSCC cells
		HN13		High OCT3 expression - ↓viability	
**Lin et al (30)**	2014	HSC3	10 mM for 48 hours	↓viability, ↑apoptosis	MOA not explored
		SAS		↔viability, ↑apoptosis	
**Madera et al (31)**	2015	CAL27	1-3 mM	↓cell proliferation, ↓colony size	↑pAMPK, ↓pS6 (mTORC1)
		CAL33			
		UMSCC47			
**Sun et al (32)**	2015	FaDu	25-100 mM for 24 hours	↓proliferation	↓miR-21-5p, ↑PDCD4 mRNA and protein
**Guimaraes et al (33)**	2016	SCC9	20 µM/ml	↓cell proliferation, ↓migration,↑apoptosis, ↑DNA fragmentation	↑PDH mRNA, ↓HIF-1α mRNA and protein, ↓HSP90, ↑caspase-3<br>Metformin inhibits HIF-1α, promotes PDH, reduces Warburg effect
		HaCat		↓cell proliferation, ↓migration	
**Harada (18)**	2016	HSC2		↓cell growth, ↑apoptosis	↓lactate, ↓HIF-1-a, ↓mTOR, ↓AKT1

		HSC3	1-10 mg/ml, 4mg/ml used for most treatment		↓lactate, ↓HIF-1-a, ↓mTOR, ↓AKT1, ↑AMPKa
		HSC4			
**Qi et al (9)**	2016	HSC3	10 µM for 48 hours	↓cell viability during hypoxia	↑pAMPK→↓NF-κB →↓HIF-1α expression and ↓HIF-1 transcription →↓GLUT1, ↓Bcl-2
**Siddappa et al (2)**	2016	Primary culture from 4NQO induced mice models	Metformin 50 µM for expression profiling (48hr), 2.5-5 µM for cell migration and clonogenic assay (12-36 hr)	Minimal effect on wound healing or colony formation	↓CSC markers (NOTCH1, STAT3), ↓CSC profile (CD44, NOTCH1, JAGGED, STAT3)
**Woo et al (11)**	2016	HN30	1-10 mM metformin for 24 hrs	↓proliferation	↓malic enzyme 2
		HN31		↓↓ proliferation	
**Chen et al (35)**	2017	SAS	5-20 mM, most treatment at 10 mM	↓ cell growth, ↓ colony formation by 50-70%, ↓ 50% BrdU incorporation ↔cell cycle distribution, ↓migration, ↓ invasion	↓LSF --> ↓Aurora-A (mRNA and protein),
		Cal27			
		SCC25			
		HGF1 (normal oral gingiva)		No change in cell growth up to 10mM. ↓ cell growth at 20mM.	MOA not explored
**Chen et al (36)**	2017	SCC4	2-20 mM Metformin for 48-72 hours	↓cell activity, ↓cell proliferation, ↓clone formation	↑G0/G1 phase, ↓S/G2 phase, ↑AMPKa, ↓p-mTOR, ↓p-S6K1, ↓p-4E-BP1
		CAL27			
**Yi et al (6)**	2017	FaDu	10-20 mM for 24-60 hrs	↔cell viability at 24 hours	↓cell adhesion molecules (integrin β4, integrin α1, fibronectin 1), ↔AMPK<br>1. ↓mTOR --> ↓ΔNp63α, ↑WWP1

**He et al (14)**	2019	HSC6	16 mM	↓colonies, ↓cell viability, ↑apoptosis	↑instrisic apoptosis, ↓ΔNp63
		HSC3			
**Inanc et al (39)**	2019	SCC131	0-16 mM (IC50: 3 mM)	↓cell viability	MOA not explored
**Lindsay et al (1)**	2019	SCC90	Metformin 5-20 mM for 72 hrs	↓proliferation	MOA not explored
		SCC152			
		SCC6			
		CAL27			
**Kuo et al (8)**	2019	JLO-1 CSC	0.25-10 mM	⟷cell proliferation	↓AKT, ↑stemness (BMI-1, CD44, Oct4, Nanog)
		HN30 ALDH+		⟷cell proliferation	↓mitochondrial complex III, ↓ROS, ↑stem cell markers
		HN30 ALDH-		↓cell proliferation	
**Wu, Yeerna et al (40)**	2019	CAL27	3 mM for 3 days	↓OCR, ↓ATP, ↓colonies, ↓spheres	Inhibit mitochrondrial complex I, ↑AMPK, ↓pS6, ↓p4EBP, ↓p70S6K
		CAL33			
		UMSCC47			
**Wu, Tang et al (12)**	2019	FaDu	8 mM for 48 hours	↓ cell viability and proliferation, ↑apoptosis	↑DNMT1 → hypermethylation of SNHG7 promoter → ↓SNHG7
**Yang et al (24)**	2019	FaDu	10 mM Metformin for 48 hrs	↓cell growth, ↑apoptosis	G1 arrest, ↓CDK1, ↓cyclin E2, ↑cleaved Caspase 3, ↑cleaved PARP

		Detroit 562			S/G2 arrest, ↓cyclin B1, ↓CDK1, ↑cleaved Caspase 3, ↑cleaved PARP
**Zhang (3)**	2019	SCC15	1-40 mM for 48 hrs (LD 10 mM)	↓proliferation, ↑apoptosis, ↓cell invasion, ↓cell migration	MOA not explored
**Zhang et al (41)**	2019	Primary cancer isolated from patients with OSCC (Passage 2-8). Normal oral fibroblasts isolated from normal gingival tissue (Passage 4-6)	0-10 mM (IC50 2.992 mM)	↓proliferation, ↑apoptosis, ↑necrosis	↑AMPK, ↓Bcl-2, ↑Bax/Bcl2, ↑cleaved-PARP, ↓mitochondrial membrane potential, ↓ATP production, ↓ROS, ↓autophagy
**He et al (15)**	2020	HSC3	16 mM	↓migration, ↓invasion	↔TWIST1, SNAI1, SNAI2, ZEB1, ↓p-STAT3, ↑E-cadherin, ↓N-cadherin
		HSC6			
**Hu et al (17)**	2020	CAL27	Metformin, IC50: 39.48 mM. 10mM used in this study	↓cell viability, ↓colony formation	Slight G0/G1, G2/M arrest, ↑p21, ↔p16, ↓p-Rb, ↓p-mTOR, ↓p-STAT3
		HSC3	Metformin, IC50: 18.32 mM. 10 mM used in this study		
		HSC6	Metformin, IC50: 6.50 mM. 1 mM used in this study		
**Huang et al (42)**	2020	Cal27	5-10 mM for 48 hrs	↓cell proliferation, ↓migration, ↑apoptosis	↑TET2, ↑5hmc, ↑cleaved PARP, ↑cleaved caspase 3
		FaDu			↑TET2, ↑5hmc
**Patil et al (43)**	2020	N/A	10-100 µM for 24-72 hrs	100µM ↓cell viability and stopped growth	↓stemness related transcription factors (OCT4, SOX2, KLF4, c-Myc, NANOG), ↓CSC expression marker CD44

**Wang et al (44)**	2020	FaDu	25 mM	↔cell proliferation	↔miR-21-5p, ↔PDCD4 mRNA
			50-100 mM	↓cell proliferation	↓miR-21-5p, ↑PDCD4 mRNA
**Zhang et al (45)**	2020	HSC3	1-4 mM for cell culture only	Lower doses (2mM) of metformin is required in cell lines with higher ITGB2 expression (HSC3, CAL33, SAS)	CAF that overexpress ITGB2 activate PI3K/AKT/mTOR, resulting in ↑lactate. Lactate oxidation requires NAD+ which produces NADH, stimulating oxidative phosphorylation. Metformin is known to inhibit mitochondrial complex I, preventing the production of NAD+.
		SAS			
		CAL27			
		SCC9			
		SCC31			
		CAL33			
**Chen et al (10)**	2021	SCC9	10 mM	↓proliferation, ↓migration, ↓healing	↔LIN28, ↓HMGA2, ↑AMPK<br>p-AMPK activates Dicer (required of processing pre-let7 to let7. Let 7 inhibits HMGA2
		CAL27			
**Hoppe-Seyler et al (46)**	2021	UDSCC2	40 µM – 5 mM	↓ viral E6 and E7 proteins	↓P-p70S6K, ↓p70S6K, ↓P-S6, ↓P-4E-BP1, ↑4E-BP1
		SCC152			
**Tsou et al (47)**	2021	FaDu	5-10 mM for 36 hrs	↓ cell viability, ↓cell proliferation, ↑apoptosis	1. ↑p38 →↓JNK → ↓STAT3 --> ↓Cyclin D1 mRNA & ↓ Bcl-2 --> cell cycle arrest (↑G1 phase) & ↑apoptosis<br>2. ↑AMPK --> ↓mTOR --> ↑p27 --> ↑LC3B, ↑Beclin 1 --> ↑autophagy<br>3. ↑MEK/ ERK/ RSK --> ↑p27 --> ↑autophagy
		Detroit 551		↔cell viability or proliferation	↑Cyclin D1 mRNA, No change in cell cycle phases

**Wang et al (23)**	2021	CAL27<br>SCC25	5-30 mM, 15 mM used for most treatment	↓proliferation, ↑apoptosis	↑G0/G1 phase, ↓S/G2 phase, ↓CDK4/ CDK6, ↑p21, ↓Bcl-2, ↑Bax<br>1. ↑Hippo pathway: ↑MST1/LATS1 --> ↑SAV1/MOB1 --> ↓YAP --> ↓mTOR --> ↓c-Myc
**Wei et al (48)**	2021	SCC-15	10 mM	↓cell proliferation, ↓colony formation	↑G1 phase, ↓G2/S phase, ↑p53, ↑p21, ↓cyclin D1, ↓a-secretase and g-secretase -->↓ proteolysis of NGFR into ICD + NGFR-N
**Yin et al (49)**	2021	CAL27	2.5-160 mM, 10 mM used for most treatment	↓proliferation, ↓migration	↓$CoCl_2$-induced EMT: ↑E-cadherin, ↓vimentin, ↓Snail1
**Chen et al(50)**	2021	FaDu<br>Detroit 562	8mM for 48 hrs	↓cell viability, ↓colony formation, ↑apoptosis	↑G0/ G1 phase,<br>↓Circ_0003214 expression → ↑miR-489-3p → ↓ADAM10
**Crist et al(57)**	2022	SCC47<br>CAL27	Co-cultured with isolated natural killer cells which have been pre-treated with 12mM of metformin for 24 hours	↑NK cytotoxicity	1. ↑perforin<br>2. ↓mTOR, ↓pSTAT3 →CXCL1 →negative feedback loop to inhibito CXCR2 receptor<br>3. ↑pSTAT1 →↑perforin
**Liu et al (52)**	2022	CAL27	2.5-40mM	↓cell viability, ↓colony formation	↑H3K27ac<br>↓DEG on axon guidance, ECM-receptor interaction, cell adhesion molecules, pluripotency stem cells, lipid metabolism pathways<br>↑DEG on TFG signalling pathway, cytokine-cytokine receptor interaction, NOD-like receptor signalling pathway, apoptosis, cell cycle, amino acid metabolism pathways

		HSC-2			↑H3K27ac<br>↓DEG on ABC transporters, peroxisome, Hippo signalling pathway, Wnt signalling pathway, ECM-receptor interaction, hormone regulation<br>↑DEG on ribosome, cytokine-cytokine receptor interaction, HIF-1 signaling pathway, amino acid metabolism
**Zhang et al (53)**	2022	SCC25	3, 5, 10mM for 48 hrs	↓cell viability	↓Cyr61 expression → ↓p-AKT
**Zhao et al(54)**	2022	CAL27	12, 24mM for 24-48 hrs	↓cell proliferation, ↓colony formation, ↓migration, ↓invasion	1. Cell cycle arrest: ↑G1 phase, ↑p21, ↓cyclin D1<br>2. Apoptotic morphology changes: cell shrinkage, nucleoplasm condensation, nuclear fragmentation<br>3. Apoptotic protein changes: ↑Bax, cleaved-caspase , ↓Bcl-2, ↓ROS<br>4. Autophagy: ↑autophagosomes, ↑LC3B-II/LC3B-I, ↑Beclin-1, ↓p62
		SCC9	3-48mM for 24-48hrs	↓cell proliferation, ↓colony formation	↑G1 phase
		SCC25			
**Ji et al(55)**	2023	CAL27	0.5-3mM for 48 hrs	↓cell proliferation, ↓cell migration	1. Exon 3 skipping of NUBP2<br>2. Exon 4 skipping of ZDHHC7<br>3. Exon 12 skipping of UL17RC<br>4. Upstream 3'ss usage of ICE2<br>5. Distal 5' ss usage of MSTO1<br>6. Reduced distal 5' ss usage of ENY2

Abbreviations: TERT, telomerase reverse transcriptase; TP53, tumour protein p53; AMPK, adenosine monophosphate-activated protein kinase; mTOR, mammalian target of rapamycin; S6K, S6 kinase; pRb, retinoblastoma protein; CDK, cyclin dependent kinase; Bcl-2, B-cell lymphoma 2; Bcl-xL, B-cell lymphoma-extra large; BAX, Bcl-2-associated X protein; CDKN2A, cyclin dependent kinase inhibitor 2A gene; EGFR, epidermal growth factor receptor; 4E-BP1, eukaryotic translation initiation factor 4E-binding protein 1; EF2, elongation factor; CDKI, cyclin dependent kinase inhibitor; ACC, acetyl-CoA carboxylase; LKB1, liver kinase B1; LN, lymph node; OCT3, organic cation transporter 3; PIK3CA, phosphatidylinositol-4,5-bisphosphate 3-kinase catalytic subunit alpha gene; MOA, mechanism of action; TMPRSS2, transmembrane protease serine 2 gene; MiR-21-5p, MicroRNA-21-5p; PDCD4, programmed cell death 4; PDH, pyruvate dehydrogenase; HIF, hypoxia-inducible factor; HSP90, heat shock protein 90; AKT, protein kinase B; NF-κB, nuclear factor kappa-light-chain-enhancer of activated B cells; GLUT1, glucose transporter 1; 4NQO, 4-nitroquinoline 1-oxide; CSC, cancer stem cells; STAT3, signal transducer and activator of transcription 3; LSF, Late SV40 factor; BMI1, B lymphoma Mo-MLV insertion region 1 homolog; Oct4, octamer-binding transcription factor 4; OCR, oxygen consumption rate; DNMT1, DNA methyltransferase 1; SNHG4, small nucleolar RNA host gene 7; OSCC, oral squamous cell carcinoma; IC50, half maximal inhibitory concentration; PARP, poly (ADP-ribose) polymerase; ROS, reactive oxygen species; SNAI, snail family transcriptional repressor; HRAS, p21 gene; TET2, Tet methylcytosine dioxygenase 2; 5hmc, 5-hydroxymethylcytosine; SOX2, SRY-box 2; KLF4, Kruppel-like factor 4; CAF, cancer associated fibroblast; NF, normal fibroblast; IHC, immunohistochemistry; ITGB2, integrin subunit beta 2; NAD, nicotinamide adenine dinucleotide; let-7, lethal 7; JNK, c-Jun N-terminal kinases; MEK, mitogen activated protein kinase; ERK, extracellular-signal-regulated kinase; RSK, ribosomal s^ kinase; MST1, macrophage stimulating 1; LATS1, large tumour suppressor kinase 1; SAV1, protein Salvador homolog 1; MOB1, Mps one binder 1; YAP, yes-associated protein; NGFR, nerve growth factor receptor; ICD, intracellular domain; CoCl2, cobalt (II) chloride; ADAM10, A disintegrin and metalloproteinase domain-containing protein; CXCL1, chemokine ligand 1; N/A, not applicable; H3K27ac, acetylation of lysine residue of histone H3 protein; DEG, differentially expressed gene; Cyr61, cysteine-rich angiogenic inducer 61; NUBP2, nucleotide binding protein 2; ZDHHC7, zinc finger

DHHC domain-containing protein 7; IL17RC, interleukin-17 receptor C; ss, splice site; ICE2, little elongation complex subunit 2; ENY2, enhance of yellow 2 transcription factor homolog; MSTO1, protein misato homolog 1.

Supplementary Table 6. The effects of metformin in combination with other treatment in HNSCC: *in vitro* studies.

Author	Year	Cell line	Metformin dose + duration	Additional therapy	Additional therapy alone	Combination Outcome	Combination mechanism
**Sandulache et al (25)**	2011	HN30	1 mM	2-DG (20 mM)	Dose-dependent ↓ in reducing potential, ↓ intraceullar lactate production, ↓ cell number	↓ cell number compared to 2-DG alone	Metformin potentiates 2-DG effects on proliferation independent on p53 status
		HN31					
**Sandulache et al (5)**	2012	HN30	10-30 mM	2-DG (5-20 mM), XRT (2-12Gy)	↓colonies	↓↓colonies compared to 2-DG alone	↑intracellular ROS, ↑AMPK, ↓mitochondiral respiration, ↓glycolysis
**Ma et al (21)**	2012	SCC9	0-25 mM for 72 hours	Gefitinib (1-25 μM) for 48 hours	N/A	Gefitinib (1-25 μM) for 48 hours	Metformin is able to act synergistically with gefitinib in LKB1 expressing cells (SCC9 and SCC25), but not LKB1 deficient cell lines (A549, HeLa)
		SCC25					
**Skinner et al (13)**	2012	HN30	5-10 mM for 24 hours	XRT (2-4.5 Gy/min) one day after seeding, and grown for 10-14 days for colony formation		Metformin shows no additional effect	↔ROS
		UMSCC 1			Radiosensitive, ↑SA-beta-gal, ↑p21, ↑ROS		
		UMSCC1 (R175H/ R282W)			Radiosensitive, ↑SA-beta-gal, ↑p21, ↑ROS		
		HN31				↓clonogenic survival, ↑senescence	↑ROS. Metformin acts as a radiosensitiser in cells with TP53 disruptive mutations

		UMSCC1 (C176F/ E336X)			More radioresistant, ↓SA-beta-gal, ↓p21, ↓ROS		
**Lin et al (30)**	2014	HSC3	10 mM for 48 hours	Dasatinib 1 μM for 24 hours	↓viability, ↑apoptosis	↓↓viability, ↑↑apoptosis	Dasatinib: ↓ERK → ↓PDK4 →↓ATP →AMPK<br>Metformin: ↑AMPK<br>Combination: AMPK →ER stress →↓EGFR
		SAS					
**Harada (18)**	2016	HSC2	1-10 mg/ml, 4 mg/ml used for most treatment	5-FU (0.5-10 μg/ml), 2.5 μg/ml used for most treatment	↓cell growth, ↑apoptosis, ↓lactate	↓↓cell growth, ↑↑apoptosis	↓↓lactate, ↓↓HIF-1-a, ↓↓mTOR, ↓↓AKT1, ↑↑AMPKa
		HSC3					
		HSC4					
**Qi et al (9)**	2016	HSC3	10 μM for 48 hours	Cisplatin 0-90 μM in hypoxic condition (1% O2)	Higher IC50 value, less apoptosis	↓IC50 for cisplatin, ↑apoptosis	Did not explore MOA of combination treatment
		SCC3					
**Siddappa et al (2)**	2016	Primary culture from 4NQO induced mice models	Metformin 50 μM for expression profiling (48 hr), 2.5-5 μM for cell migration and clonogenic assay (12-36 hr)	Curcumin 10 μM	↓CSC profile (CD44, CD133, NOTCH1, SOX3, JAGGED)	↓in wound healing and colony formation	↓CSC markers (NOTCH1, STAT3)<br>↓CSC profile (CD44, CD133, NOTCH1, ALDH1A1, JAGGED1, STAT3)
**Yi et al (6)**	2017	FaDu	10-20 mM for 24-60 hours	2-DG(20 mM)	↔cell vaibility at 24 hours, ↔ΔNp63α	↓cell viability	↓ΔNp63α, ↑PARP1
**Yin et al (20)**	2018	HSC3	100 μM	Gefitinib (1 μM) in hypoxic condition (1% O2)	↑cyclin D1 mRNA and protein, ↑p-AKT, ↔p-ERK	↓cell growth, ↑apoptosis	↓cyclin D1 miRNA and protein, ↑G1 phase, ↓EMT (↑E-cadherin, ↓vimentin, ↓Slug, ↓α-SMA), ↓HIF-1α, ↓p-AKT

		HN4			↑cyclin D1 mRNA and protein, ↑p-AKT, ↔p-ERK		↓cyclin D1 miRNA and protein, ↑G1 phase, ↓EMT (↑E-cadherin, ↓vimentin, ↓Twist, ↓α-SMA), ↓HIF-1α, ↓p-AKT
**He et al (14)**	2019	HSC6	16 mM	4SC-202 (0.4 µM)	↓colonies, ↓cell viability, ↓ΔNp63	↓↓colonies, ↓↓survival fraction, ↑↑apoptosis, ↓↓ΔNp63	↑instrinc apoptosis markers (P53, Bax, cleaved caspase-9, cleaved caspase-3, cleaved PARP), ↓Bcl-2. No change in caspase-8
		HSC3					
**Inanc et al (39)**	2019	SCC131	0-16 mM (IC50: 3 mM)	Dichloroacetate (15-120 mM, IC50: 23 mM)	↓cell viability	When combined at a ratio of 1:60, has inhibitory effect values of 0.05-0.97, with combination index ranging from 0.77-0.81	MOA not explored
**Kuo et al (8)**	2019	JLO-1 CSC	0.25-10 mM	Cisplatin 10-20 µM	↓cell proliferation, ↑DNA strand breaks	↔cell proliferation	↓DNA strand breaks
		HN30 ALDH+			↓cell proliferation	↔cell proliferation	MOA not explored
		HN30 ALDH-			↓cell proliferation	↓cell proliferation	
**Lindsay et al (1)**	2019	SCC90	Metformin 5-20 mM for 72 hours	Curcumin 100-200 µM	↓ proliferation ↑apoptosis	100 µM curcumin+10mM metformin ↓proliferation, ↑apoptosis (equivalent to 100 µM curcumin)	Did not explore MOA
		SCC152					
		SCC6					
		CAL27					
**Wu, Tang et al (12)**	2019	FaDu	8 mM for 48 hours	Taxol 40 nM; XRT (2-6 Gy/min)	Taxol alone: ↓ cell viability, ↑apoptosis	↓IC50 for taxol, ↓↓cell viability, ↑↑apoptosis	↓SNHG7 --> ↓Drug-resistant genes expression (MDR1, MRP7, LRP, TRAG3)

**Yang et al (24)**	2019	FaDu	10 mM Metformin for 48 hours	BPTES 20 µM for 48 hours	↓cell growth, ↑apoptosis, G2 arrest, ↓cyclin E2, ↑p21, ↑cleaved Caspase 3, ↑cleaved PARP	↓↓cell number, ↓↓cell viability	↓cyclin B1, ↓ CDK1, ↓cyclin E2, ↑p21, ↑cleaved Caspase 3, ↑cleaved PARP
		Detroit 562			↓cell growth, ↑apoptosis, S phase arrest, ↑p21, ↑cleaved Caspase 3, ↑cleaved PARP		↓cyclin B1, ↓CKD1, ↑p21, ↑cleaved Caspase 3, ↑cleaved PARP
**Yin et al (19)**	2019	CAL27	10 mM for 24-48 hours	Gefitinib (corresponding IC50 for each cell line)	N/A	↓gefitinib resistance	↓CCL15 secretion by macrophages, ↓CCR1 receptor on tumour cells,, ↓IGFBP-4, ↓NT-4
		SCC7					
		FaDu					
		SCC9					
		JHU011					
**Zhang et al (3)**	2019	SCC15	1-40 mM for 48 hrs (LD 10 mM)	Adriamycin (0.01-1 mg/L) (LD 0.05 mg/L)	↓proliferation, ↑apoptosis, ↓cell invasion, ↓cell migration	0.05mg/ml adriamycin + 10mM metformin ↓↓proliferation, ↑↑apotosis, ↓↓cell invasion, ↓↓cell migration	MOA not explored
**He et al (15)**	2020	HSC3	16 mM	4SC-202 (0.4 µM)	↓migration, ↓invasion	↓↓migration, ↓↓invasion	↓↓p-STAT, ↓↓TWIST1 which then leads to reduction of EMT (↑E-cadherin, ↓N-cadherin)
		HSC6					
**Hu et al (17)**	2020	CAL27	Metformin, IC50: 39.48 mM. 10mM used in this study	LY2835219, IC50: 1.25 µM	↓cell viability, ↓colony formation. ↑p16, ↑p21, ↓pRb, ↑p-mTOR, ↑p-STAT3, ↑CSC	Metformin (10mM) + varying doses of LY2835219	Significant G0/G1 arrest, reduced S, G2/M phase, ↑↑p21, ↔p16, ↓p-Rb, ↓tumour promoting cytokines (IL6, IL8, MCP1, GRO), maintain

		HSC3	Metformin, IC50: 18.32 mM. 10 mM used in this study	LY2835219, IC50: 0.1 μM	(ALDH1A1, CD44, Nanog). Significant G0/G1 arrest, reduced S, G2/M phase.	↓↓cell viability, ↓↓colony formation, ↓sphere formation	senescence cytokines (IL1α, IL1β, TGFβ-, CCL5), ↓CSC (ALDH1A1, CD44, Nanog), ↓p-mTOR, ↓p-STAT3
		HSC6	Metformin, IC50: 6.50 mM. 1 mM used in this study	LY2835219, IC50: 0.3μM			
**Song et al (7)**	2020	HSC3	0.001953 - 1.25 mM	Cisplatin (0.1953 - 6.25 mM)	Reduction in cell viability	Reduction in cell viability with strong synergistic effect when metformin:cisplatin ratio = 1:10 regardless of normoxia/ hypoxia environment	MOA not explored
		FaDu					
**Chen et al (10)**	2021	SCC9	10 mM	C1632 (60 μM and 240 μM)	↓LIN28, ↓HMGA2	↓↓proliferation, ↓↓healing, ↓↓migration	↓↓LIN28, ↓↓HMGA2, ↑↑AMPK, ↑↑p-AMPK; C1632 inhibits LIN28 which allows maturation of let7. Metformin promotes maturation of let 7 by activating dicer.
		CAL27				↓↓proliferation, no difference in migration compared to single treatment	
**Wang et al (23)**	2021	CAL27	5-30 mM, 15 mM used for most treatment	Verteporfin, 1 μM	↓CDK4/ CDK6, ↑p21, ↓Bcl-2, ↑Bax	↑↑apoptosis	↑↑G1 phase, ↓CDK4/ CDK6, ↑p21, ↓Bcl-2, ↑Bax, ↓mTOR, ↓p-mTOR, ↓c-Myc
		SCC25					
**Zhao et al(54)**	2022	CAL27	12, 24 mM for48 hrs	Hydroxychloroquine, 20 μM	↔apoptosis,	↑↑apoptosis,	↑Bax, ↓Bcl-2
		SCC9			↔apoptosis	↑↑apoptosis	
		SCC25			↑apoptosis	↑↑apoptosis	

Abbreviations: 2-DG, 2-deoxyglucose; XRT, radiotherapy; ROS, reactive oxygen species; AMPK, AMP activated protein kinase; ERK, extracellular signal-regulated kinase; PDK4, pyruvate dehydrogenase kinase 4; 5-FU, 5-fluorouracil; HIF, hypoxia inducible factor; mTOR, mammalian target of rapamycin; AKT, protein kinase B; IC50, half-maximal inhibitory concentration; 4NQO, 4-nitroquinoline 1-oxide; CSC, cancer stem cell; EMT, epithelial mesenchymal transition; α-SMA, alpha-smooth muscle actin; BAX, Bcl-2-associated X protein; Bcl-2, B-cell lymphoma 2; MOA, mechanism of action; DNA, deoxyribonucleic acid; SNHG7, small nucleolar RNA host gene 7; MDR1, multi-drug resistance gene; MRP7, multidrug resistance protein 7; LRP, lung resistance-related protein; TRAG3, taxol-resistance-associated gene-3; BPTES, bis-2-ethyl sulfide; CDK, cyclin dependent kinase; CCL15, CC-motif chemokine ligand 15; CCR1, CC-motif chemokine receptor; IGFBP-4, insulin like growth factor binding protein 4; NT-4, neurotrophin-4; STAT, signal transducer and activator of transcription; Rb, retinoblastoma protein; IL, interleukin; HMGA2, high-mobility group AT-hook 2; let 7, lethal 7.

Supplementary Table 7. Metformin in HNSCC: *in vivo* studies

Author	Year	Animal model	Induced/ xenograft	Treatment groups	Combination therapy	Outcome	Mechanism
**Luo et al (26)**	2012	4 weeks Balb/c nude mice	CAL27 injected into the back	Drinking water for 15 days<br>1. Metformin: 200 µg/ml<br>2. water	N/A	Metformin treatment group: ↓tumour growth, ↓weight of tumour,	↓cyclin D1 (IHC), ↑apoptotic cells(TUNEL)
**Vitale-Cross et al (28)**	2012	4-6 week old C57BL/6 mice	4NQO (50 µg/ml for 8, 10, 12, 14, 16 weeks) then water until week 22	After 14 weeks, for 8 weeks<br><br>1. Metformin (50 mg/kg/day), IP injection<br><br>2. Equal volume saline, IP injection	N/A	↓number and size of oral lesions, ↓progression of premalignant lesions to malignant	↓pS6 (therefore mTOR) in the basal and suprabasal cell layers
**Skinner et al (13)**	2012	8-12 weeks athymic male mice	HN31 cells injected into anterior half of oral tongue	Once tumour growth noted, tumours were treated for 8 days with<br>1. Radiation (5 Gy)<br>2. Metformin (250 mg/kg/day), IP injection<br>3. Radiation + Metformin	XRT	↓tumour growth in combination group compared to either treatment alone.	MOA not explored
**Lin et al (30)**	2014	6 weeks old NCr athymic female nude mice	HSC3 and SAS cells injected S/C into rear left flank	After tumour reach 100 mm^3, treatment for 5 days/week for 4 weeks with<br>1. Metformin (400 mg/kg) orally<br>2. Dasatinib (60 mg/kg/day) orally<br>3. Combination	Dasatinib	Tumour volume: Control > Metformin > Dasatinib > Combination	↑p-AMPK, ↑p-eIF2a, ↓EGFR

**Madera et al (31)**	2015	4-6 week old nude mice	CAL27, CAL33 or UMSCC47 cells injected S/C in flanks; MOA explored in mice with UMSCC47 tumours	After 1 day, oral treatment of<br>1. Water<br>2. Metformin (2.5 mg/ml - equivalent to 2 µg/ml found in human plasma)	N/A	↓ tumour size and weight, ↔body weight, ↓fraction of proliferation cells (BrdUrd)	↓pS6, ↑ non-phosphorylated 4E-BP1
**Harada et al (18)**	2016	4 weeks BALB/c athymic nude mice	HSC2 cells injected into S/C tissue	4 weeks of treatment:<br>1. sterile saline<br>2. 5-FU (10 mg/kg), IP injection<br>3. Metformin (200 mg/kg), IP injection<br>4. 5-FU + Metformin	5-FU	Combination group: ↓↓tumour growth	Combination groups had: ↓HIF-1-a, ↓mTOR, ↓AKT1, ↑↑AMPKa, ↑↑apoptosis<br>Metformin single treatment: ↓HIF-1-a, ↓mTOR, ↓AKT1, ↑AMPKa, ↑apoptosis<br>5-FU single treatment: ↓AKT1, ↑AMPKa, ↑apoptosis
**Qi et al (9)**	2016	Immunocompromised nude mice	HSC3 injected into right flank	After 10 days, injected every 3 days for 3 weeks<br>1. PBS<br>2. Cisplatin (10 mg/kg), IP injection<br>3. Metformin (10 mg/kg), IP injection<br>4. Cisplatin + Metformin	Cisplatin (10mg/kg)	↓tumour volume in cisplatin alone and ↓↓ tumour volume in combination group; ↑TUNEL positive in combination group	↓p-p65 (NF-κβ) →↓HIF-1α→↓GLUT1, ↓Bcl-2
**Siddappa et al (2)**	2016	4-6 week C57BL/6 female mice	4NQO (50 µg/ml) in drinking water for 17 weeks	Drinking water for 8 weeks<br>1. Water<br>2. Metformin: 5 mg/ml/week<br>3. Curcumin 1g/70g body weight/ week<br>4. Combiantion of metformin and curcumin	Curcumin	↓number of lesions and ↓ size of lesions in metformin alone, curcumin alone and combination	↓ NF-kB in curcumin and combination<br>↓ pS6 in metformin and combination<br>↓in NOTCH1, STAT3 in metformin and combination<br>↓in CD133 in combination group
**Chen et al (35)**	2017	Male nude mice	SAS, SCC25 cells injected into flanks	SAS, SCC25 pre-treated with PBS or metformin (5 mM) for 2 weeks	N/A	Metformin group ↓tumour volume and mass, ↓PCNA and Ki67 expression	↓Aurora-A

		6 week female nude mice	SAS, SCC25 cells injected into the tail veins			Metformin group ↓pulmonary metastatic nodules	MOA not explored
**Thompson et al (34)**	2017	8 weeks Fascher 344 male rats	4NQO (20 ppm, equiv to 0.105 mM) in drinking water for 10 weeks	1. Standard Purina 5001 Lab diet<br>2. 250ppm Metformin, starting one day after 4NQO ceased<br>3. 500ppm Metformin, starting one day after 4NQO ceased<br>4. 500ppm Metformin, starting 6 weeks after 4NQO ceased<br>(250ppm metformin = 1.94 mM<br>500 ppm metformin = 3.87 mM)	N/A	The incidence of OSCC development, invasion score, incidence of preneoplastic lesions did not differ significantly when compared to the control group.	MOA not explored
**Yi et al (6)**	2017	5 weeks female BALB/c nude mice	FaDu cells injected S/C into right scruff	After day 16, every 2 days, for 32 days,<br>1. Control (Saline), IP injection<br>2. Metformin (400 mg/kg), IP injection<br>3. 2-DG (400 mg/kg), IP injection<br>4. Metformin (400 mg/kg) and 2-DG (400 mg/kg), IP injection	2-DG	↓tumour weight and volume	↓p63 expression
**Tassone et al (38)**	2018	4-6 weeks female athymic nude mice	Injection into bilateral flanks<br>1. CAL27<br>2. CAL27 + BJI<br>3. BJI	7 days after injection:<br>1. Metformin: 1mg/ml (6 ml/day; equiv to 300 mg/kg/day)<br>2. Water	Fibroblasts (BJI)	CAL27+BJI group: ↓tumour volume with metformin	↓MCT1, ↑cancer apoptotic nuclei, ↑stromal CAV1, ↔MCT4

			Injection into bilateral flanks<br>1. CAL27 + BJI<br>2. CAL27 + BJI (with CAV1 knockdown)			CAL27 + BJI (with CAV1 knockdown) group: ⟷tumour volume	CAV1 essential for metformin's action
**Verma et al (37)**	2018	8-12 weeks female CB.17 severe combined immunodeficient mice	FaDu cells injected into the floor of the mouth via transcervical approach	After 10 days, treatment with<br>1. Metformin (200mg/kg), IP, 5 days<br>2. Control (water), IP, 5 days	N/A	↓tumour volume, ↓proliferation marker (Ki-67), ⟷endothelial marker (CD31)	↑tumour oxygen saturation, ↑total haemoglobin, ⟷2-DG uptake
**Yin et al (20)**	2018	BALB/cJNju-Foxn1nu/Nju	30 µL HSC3 injected into tongue	After 10 days, daily oral gavage for 3 weeks,<br>1. PBS<br>2. Gefitinib (60 mg/kg)<br>3. Metformin (200 mg/kg)<br>4. Gefitinib + Metformin	Gefitinib	↑body weight and ↓tumour volume in the combination treatment groups.	↓HIF-1α, ↓cyclin D1, ↓Ki-67, ↓EMT (↑E-cadherin, + TUNEL staining)
**He et al (14)**	2019	4-6 week BALB/c nude female mice	HSC6 injected into right forelimb	Treatment for 25 days<br>1. control (Solvent)<br>2. metformin (100 mg/kg), IP<br>3. 4SC-202 (80 mg/kg), intragastric<br>4. combination of metformin and 4SC-202	4SC-202	Significant reduction in tumour volume and weight in combination group compared to single agent alone	↑cell apoptosis rate (combination > single agent),
			4NQO (50 µg/ml for 16 weeks, then water for 6 weeks)	1. control (Solvent)<br>2. Combination: metformin (100 mg/kg) IP + 4SC-202 (80 mg/kg) intragastric<br>4. cisplatin (1 mg/kg) IP		Suppression of progression of oral carcinoma, ↓area of tongue lesions (more reduced than cisplatin), ↓dysplasia, ↓SCC, ↓ΔNp63	4SC-202 + metformin combination: ↓ΔNp63 protein via ↑ubiquitination.<br>Cisplatin: ↓ΔNp63 mRNA and protein

**Yin et al (19)**	2019	C3H/HeNCrl mice	SCC7 cells injected S/C into dorsal flank	5 days after injection, for 21 days<br>1. PBS orally<br>2. Gefitinib (60 mg/kg), orally<br>3. Metformin (200 mg/kg), orally<br>4. Gefitinib + metformin	Gefitinib	Combination group ↓ tumour volume compared to single agent. ↓Ki67	↓M2 macrophages, ↓CCL9 (homologous to human CCL15), ↓CCR1, ↓NF-κB translocation
**Wu, Yeerna et al (40)**	2019	4-6 week old nude mice	CAL33 cells injected S/C into flanks	1. Control (Water)<br>2. Metformin orally (2.5 mg/ml)	N/A	↓tumour weight and volume, ↓Ki-67	↓pS6, ↓pS6K, ↓p4E-BP1, ↑pAMPK, ↑pACC, ↓CSC markers (CD44, CD133, ALDH1A1, BMI1, SOX2, ↑ squamous differentiation (keratinisation, CK10)
**Wu, Tang et al (12)**	2019	2 months old nude mice	FaDu cells injected into left rib	FaDu cells (with or without overexpression of SNHG7) treated with metformin	N/A	Tumour size: Control > Metformin+SNHG7 > Metformin	↑p-AMPK, ↓SNHG7 expression
**Hu et al (17)**	2020	Nude mice	HSC6 injected into right armpit	3 weeks of<br>1. Vehicle control<br>2. LY2835219 (25 mg/kg/day), orally<br>3. Metformin (100 mg/kg/day), IP<br>4. LY2835219 + Metformin	LY2835219 (Ademaciclib)	Single agent did not demonstrate significant difference. Combination group ↓tumour volume and weight,↓Ki67, ↓PCNA	Combination treatment group: ↑p21, ↓pRb, ↓CD44, ↓ALDH1A1
			Patient derived xenograft, P2 xenografts used, tumour grown to 100 mm^2 prior to starting treatment	3 weeks of<br>1. Vehicle control<br>2. LY2835219 (40 mg/kg/day), orally<br>3. Metformin (200 mg/kg/day), IP<br>4. LY2835219 + Metformin		Single agent did not demonstrate significant difference. Combination group ↓tumour volume and weight,↓Ki67, ↓PCNA	
			CAL27 cells incubated with complete medium from different groups for 96 hours, then injected subcutaneously into right	Cells pre-treated with complete media of previous cell treatments:<br>1. Control<br>2. LY2835219		More tumour formation in LY complete medium than control or LY+Met group	↑CD44, ↑ALDH1a1 in LY complete medium group

			armpit to assess tumorigenesis	3. Metformin<br>4. LY2835219 + Metformin			
**Song et al (7)**	2020	6 weeks Balb/c male nude mice	Xenograft (HSC3) injected into back	7 groups, intra-tumoural injection with<br>1. Saline only<br>2. Cisplatin only<br>3. Metformin only<br>4. Cisplatin + metformin<br>5. Ce6 (chlorin e6) nanoparticles + laser<br>6. CECMa (Ce6-PEG-cisplatin-metformin) only<br>7. CECMa + laser	Cisplatin (10mg/kg), Cisplatin:metformin ratio 10:1	CECMa + laser showed tumour elimiation, ↓Ki-67 staining, ↑**γ**-H2AX, ↑Hsp 70	Cisplatin causes DNA double-strand breaks. Metformin enhances this effect.
**Chen et al(10)**	2021	6-8 weeks BaALB/C male nude mice	Xenograft (CAL27) injected into back	1. Drinking sterile water<br>2. Metformin (250mg/kg/day)<br>3. C1632 (40 mg/kg for 3 days)<br>4. C1632 (40 mg/kg for 3 days) + metformin (250 mg/kg/day; IP for 18 days)	C1632 (40mg/kg) for 3 days	↓tumour weight and size in combination group compared to control or single agent	↓LIN28, ↓HMGA2, ↑↑AMPK
**Yin et al (49)**	2021	4-6 weeks male BALB/C nude mice	CAL27 cells injected S/C into right flank	CAL27 cells cultured with 300µM of $CoCl_2$ with or without 10 mM Metformin for 48 hours prior to S/C<br><br>injection	N/A	↓tumour weight and volume	MOA not explored
**Chen et al (50)**	2021	6-8 weeks female BALB/C nude mice	FaDu cells with or without circ_0003214 overexpression injected S/C	27 days<br><br>1. FaDu cells alone<br>2. FaDu cells + metformin treatment (IP)	N/A	↓tumour weight and volume	↓circ_0003214, ↓ADAM10 mRNA and protein

				3. FaDu cells + circ_0003214 + metformin treatment			
**Liu et al (52)**	2022	6 weeks BALB/C nude mice	CAL27 cells injected into right armpit	200 µg/ml of metformin added to drinking water for 3 weeks	N/A	↓tumour weight and volume, ↓Ki67	No stable effect on expression of H3K27me3 and H3K27ac
**Zhao et al(54)**	2022	5-6 weeks female nude mice	CAL27 injected S/C	18 days<br>1. PBS<br>2. Metformin (250mg/kg/day, IP)<br>3. Hydroxychloroquine (50mg/kg/day, IP)<br>4. Metformin + hydroxychloroquine	Hydroxychloroquine	↓tumour weight and size in combination group compared to control or single agent	↓Ki-67, ↑cleaved-caspase 3 in metformin group and further more in combination group

Abbreviations: IHC, immunohistochemistry; TUNEL, Terminal deoxynucleotidyl transferase dUTP nick end labelling; 4NQO, 4-nitroquinoline 1-oxide; XRT, radiotherapy; MOA, mechanism of action; S/C, subcutaneous; IP, intraperitoneal; AMPK, AMP-activated protein kinase; eIF2a, eukaryotic translation initiation factor 2A; EGFR, epidermal growth factor receptor; N/A, not applicable; BrdUrd, bromodeoxyuridine; pS6, ribosomal protein S6; 4E-BP1, eukarotic translation initiation factor 4E-binding protein 1; 5-FU, 5-fluorouracil; HIF, hypoxia inducible factor; mTOR, mammalian target of rapamycin; AKT, protein kinase B; PBS, phosphate-buffered saline; GLUT1, glucose transporter 1; Bcl-2, B-cell lymphoma 2; NF-kB, nuclear factor kappa B; PCNA, proliferating cell nuclear antigen; ppm, parts per million; 2-DG, deoxyglucose; BJI, human fibroblast; CAV1, caveolin-1; MCT, mnocarboxylate transporters; EMT, epithelial-mesenchymal transition; CCL, CC-motif chemokine ligand; CCR1, CC-motif chemokine receptor; ACC, acetyl-CoA carboxylase; CSC, cancer stem cell; ALDH, aldehyde dehydrogenase; BMI1, B lymphoma Mo-MLV insertion region 1 homolog); SNHG7, small nucleolar RNA host gene 7; HSP70, heat shock protein 70; $CoCl_2$, cobalt (II) chloride; HMGA2, high-mobility group AT-hook 2.

1. Lindsay C, Kostiuk M, Conrad D, O'Connell DA, Harris J, Seikaly H, et al. Antitumour Effects of Metformin and Curcumin in Human Papillomavirus Positive and Negative Head and Neck Cancer Cells. *Molecular Carcinogenesis* (2019) 58(11):1946-59.
2. Siddappa G, Kulsum S, Ravindra DR, Kumar VV, Raju N, Raghavan N, et al. Curcumin and Metformin-Mediated Chemoprevention of Oral Cancer Is Associated with Inhibition of Cancer Stem Cells. *Molecular Carcinogenesis* (2017) 56(11):2446-60.
3. Zhang J. Effect of Adriamycin Combined with Metformin on Biological Function of Human Tongue Cancer Ssc-15 Cells. *Oncology Letters* (2019) 17(6):5674-80.
4. Sandulache VC, Hamblin JS, Skinner HD, Kubik MW, Myers JN, Zevallos JP. Association between Metformin Use and Improved Survival in Patients with Laryngeal Squamous Cell Carcinoma. *Head & Neck* (2014) 36(7):1039-43.
5. Sandulache VC, Skinner HD, Ow TJ, Zhang A, Xia X, Luchak JM, et al. Individualizing Antimetabolic Treatment Strategies for Head and Neck Squamous Cell Carcinoma Based on Tp53 Mutational Status. *Cancer* (2012) 118(3):711-21.
6. Yi Y, Chen D, Ao J, Sun S, Wu M, Li X, et al. Metformin Promotes Amp-Activated Protein Kinaseindependent Suppression of Deltanp63alpha Protein Expression and Inhibits Cancer Cell Viability. *Journal of Biological Chemistry* (2017) 292(13):5253-61. doi: http://dx.doi.org/10.1074/jbc.M116.769141.
7. Song C, Tang C, Xu W, Ran J, Wei Z, Wang Y, et al. Hypoxia-Targeting Multifunctional Nanoparticles for Sensitized Chemotherapy and Phototherapy in Head and Neck Squamous Cell Carcinoma. *International Journal of Nanomedicine* (2020) 15:347-61.
8. Kuo SZ, Honda CO, Li WT, Honda TK, Kim E, Altuna X, et al. Metformin Results in Diametrically Opposed Effects by Targeting Non-Stem Cancer Cells but Protecting Cancer Stem Cells in Head and Neck Squamous Cell Carcinoma. *International Journal of Molecular Sciences* (2019) 20(1):07.
9. Qi X, Xu W, Xie J, Wang Y, Han S, Wei Z, et al. Metformin Sensitizes the Response of Oral Squamous Cell Carcinoma to Cisplatin Treatment through Inhibition of Nf-Kappab/Hif-1alpha Signal Axis. *Scientific Reports* (2016) 6:35788.
10. Chen H, Sa G, Li L, He S, Wu T. In Vitro and in Vivo Synergistic Anti-Tumor Effect of Lin28 Inhibitor and Metformin in Oral Squamous Cell Carcinoma. *European Journal of Pharmacology* (2021) 891:173757.
11. Woo SH, Yang LP, Chuang HC, Fitzgerald A, Lee HY, Pickering C, et al. Down-Regulation of Malic Enzyme 1 and 2: Sensitizing Head and Neck Squamous Cell Carcinoma Cells to Therapy-Induced Senescence. *Head & Neck* (2016) 38 Suppl 1:E934-40.
12. Wu P, Tang Y, Fang X, Xie C, Zeng J, Wang W, et al. Metformin Suppresses Hypopharyngeal Cancer Growth by Epigenetically Silencing Long Non-Coding Rna Snhg7 in Fadu Cells. *Frontiers in Pharmacology* (2019) 10(feburay). doi: http://dx.doi.org/10.3389/fphar.2019.00143.
13. Skinner HD, Sandulache VC, Ow TJ, Meyn RE, Yordy JS, Beadle BM, et al. Tp53 Disruptive Mutations Lead to Head and Neck Cancer Treatment Failure through Inhibition of Radiation-Induced Senescence. *Clinical Cancer Research* (2012) 18(1):290-300.
14. He Y, Tai S, Deng M, Fan Z, Ping F, He L, et al. Metformin and 4sc-202 Synergistically Promote Intrinsic Cell Apoptosis by Accelerating Deltanp63 Ubiquitination and Degradation in Oral Squamous Cell Carcinoma. *Cancer Medicine* (2019) 8(7):3479-90.
15. He Y, Fan Z, He L, Zhang C, Ping F, Deng M, et al. Metformin Combined with 4sc-202 Inhibited the Migration and Invasion of Oscc Via Stat3/Twist1. *OncoTargets and therapy* (2020) 13:11019-29.

16. Inanc S, Keles D, Sipahi M, Baskin Y, Oktay G. Effects of Metformin and Dichloroacetate on Mitochondrial Energy Metabolism in Oral Cavity Cancer Cells. *ENT Updates* (2018) 9(2):68-73. doi: http://dx.doi.org/10.1002/2211-5463.12453.
17. Hu Q, Peng J, Jiang L, Li W, Su Q, Zhang J, et al. Metformin as a Senostatic Drug Enhances the Anticancer Efficacy of Cdk4/6 Inhibitor in Head and Neck Squamous Cell Carcinoma. *Cell Death & Disease* (2020) 11(10):925.
18. Harada K, Ferdous T, Harada T, Ueyama Y. Metformin in Combination with 5-Fluorouracil Suppresses Tumor Growth by Inhibiting the Warburg Effect in Human Oral Squamous Cell Carcinoma. *International Journal of Oncology* (2016) 49(1):276-84.
19. Yin X, Han S, Song C, Zou H, Wei Z, Xu W, et al. Metformin Enhances Gefitinib Efficacy by Interfering with Interactions between Tumor-Associated Macrophages and Head and Neck Squamous Cell Carcinoma Cells. *Cellular Oncology* (2019) 42(4):459-75.
20. Yin X, Wei Z, Song C, Tang C, Xu W, Wang Y, et al. Metformin Sensitizes Hypoxia-Induced Gefitinib Treatment Resistance of Hnscc Via Cell Cycle Regulation and Emt Reversal. *Cancer management and research* (2018) 10:5785-98.
21. Ma L, Niknejad N, Gorn-Hondermann I, Dayekh K, Dimitroulakos J. Lovastatin Induces Multiple Stress Pathways Including Lkb1/Ampk Activation That Regulate Its Cytotoxic Effects in Squamous Cell Carcinoma Cells. *PLoS One* (2012) 7(9):e46055. Epub 2012/10/03. doi: 10.1371/journal.pone.0046055.
22. Lin YCL, Wu MH, Wei TT, Lin YC, Chen CC. Ampk Activation Mediates Dasatinib-Induced Egfr Degradation and Apoptosis in Head and Neck Cancer. *Cancer Research Conference: AACR Special Conference on Cellular Heterogeneity in the Tumor Microenvironment* (2014) 75(1 SUPPL. 1). doi: http://dx.doi.org/10.1158/1538-7445.CHTME14-A73.
23. Wang Y, Zhang Y, Feng X, Tian H, Fu X, Gu W, et al. Metformin Inhibits Mtor and C-Myc by Decreasing Yap Protein Expression in Oscc Cells. *Oncology Reports* (2021) 45(3):1249-60.
24. Yang J, Guo Y, Seo W, Zhang R, Lu C, Wang Y, et al. Targeting Cellular Metabolism to Reduce Head and Neck Cancer Growth. *Scientific Reports* (2019) 9(1):4995.
25. Sandulache VC, Ow TJ, Pickering CR, Frederick MJ, Zhou G, Fokt I, et al. Glucose, Not Glutamine, Is the Dominant Energy Source Required for Proliferation and Survival of Head and Neck Squamous Carcinoma Cells. *Cancer* (2011) 117(13):2926-38.
26. Luo Q, Hu D, Hu S, Yan M, Sun Z, Chen F. In Vitro and in Vivo Anti-Tumor Effect of Metformin as a Novel Therapeutic Agent in Human Oral Squamous Cell Carcinoma. *BMC Cancer* (2012) 12:517.
27. Sikka A, Kaur M, Agarwal C, Deep G, Agarwal R. Metformin Suppresses Growth of Human Head and Neck Squamous Cell Carcinoma Via Global Inhibition of Protein Translation. *Cell Cycle* (2012) 11(7):1374-82.
28. Vitale-Cross L, Molinolo AA, Martin D, Younis RH, Maruyama T, Patel V, et al. Metformin Prevents the Development of Oral Squamous Cell Carcinomas from Carcinogen-Induced Premalignant Lesions. *Cancer Prevention Research* (2012) 5(4):562-73.
29. Patel H, Younis RH, Ord RA, Basile JR, Schneider A. Differential Expression of Organic Cation Transporter Oct-3 in Oral Premalignant and Malignant Lesions: Potential Implications in the Antineoplastic Effects of Metformin. *Journal of Oral Pathology & Medicine* (2013) 42(3):250-6.

30. Lin YC, Wu MH, Wei TT, Lin YC, Huang WC, Huang LY, et al. Metformin Sensitizes Anticancer Effect of Dasatinib in Head and Neck Squamous Cell Carcinoma Cells through Ampk-Dependent Er Stress. *Oncotarget* (2014) 5(1):298-308.
31. Madera D, Vitale-Cross L, Martin D, Schneider A, Molinolo AA, Gangane N, et al. Prevention of Tumor Growth Driven by Pik3ca and Hpv Oncogenes by Targeting Mtor Signaling with Metformin in Oral Squamous Carcinomas Expressing Oct3. *Cancer Prevention Research* (2015) 8(3):197-207.
32. Sun R, Ma X, Cai X, Pan X, Liu D. The Effect and Mechanism of Action of Metformin on in Vitro Fadu Cell Proliferation. *Journal of International Medical Research* (2016) 44(5):1049-54.
33. Guimaraes TA, Farias LC, Santos ES, de Carvalho Fraga CA, Orsini LA, de Freitas Teles L, et al. Metformin Increases Pdh and Suppresses Hif-1alpha under Hypoxic Conditions and Induces Cell Death in Oral Squamous Cell Carcinoma. *Oncotarget* (2016) 7(34):55057-68.
34. Thompson MD, Lubet RA, McCormick DL, Clapper ML, Bode AM, Juliana MM, et al. Lack of Chemopreventive Efficacy of Metformin in Rodent Models of Urinary Bladder, Head and Neck, and Colon/Intestine Cancer. *Oncology Letters* (2017) 14(3):3480-6.
35. Chen CH, Tsai HT, Chuang HC, Shiu LY, Su LJ, Chiu TJ, et al. Metformin Disrupts Malignant Behavior of Oral Squamous Cell Carcinoma Via a Novel Signaling Involving Late Sv40 Factor/Aurora-A. *Scientific Reports* (2017) 7(1):1358.
36. Chen Y, Liao L, Qiu J, Zheng Y, Tang S, Li R, et al. Research on the Inhibitory Effect of Metformin on Human Oral Squamous Cell Carcinoma Scc-4 and Cal-27 Cells and the Relevant Molecular Mechanism. *Biomedical Research (India)* (2017) 28(14):6350-4.
37. Verma A, Rich LJ, Vincent-Chong VK, Seshadri M. Visualizing the Effects of Metformin on Tumor Growth, Vascularity, and Metabolism in Head and Neck Cancer. *Journal of Oral Pathology & Medicine* (2018) 47(5):484-91.
38. Tassone P, Domingo-Vidal M, Whitaker-Menezes D, Lin Z, Roche M, Tuluc M, et al. Metformin Effects on Metabolic Coupling and Tumor Growth in Oral Cavity Squamous Cell Carcinoma Coinjection Xenografts. *Otolaryngology - Head & Neck Surgery* (2018) 158(5):867-77.
39. Inanc S, Keles D, Eskiizmir G, Baskin Y, Oktay G. Metformin and Dichloroacetate Combination Exert a Synergistic Effect on Cell Viability of Oral Squamous Cell Carcinoma. *ENT Updates* (2019) 9((2)):68-73.
40. Wu X, Yeerna H, Goto Y, Ando T, Wu VH, Zhang X, et al. Metformin Inhibits Progression of Head and Neck Squamous Cell Carcinoma by Acting Directly on Carcinoma-Initiating Cells. *Cancer Research* (2019) 79(17):4360-70.
41. Zhang Z, Liang X, Fan Y, Gao Z, Bindoff LA, Costea DE, et al. Fibroblasts Rescue Oral Squamous Cancer Cell from Metformin-Induced Apoptosis Via Alleviating Metabolic Disbalance and Inhibiting Ampk Pathway. *Cell Cycle* (2019) 18(9):949-62.
42. Huang R, Ge H, Wang D, Wang Y, Zhang W, Yang J, et al. Restoration of Tet2 Deficiency Inhibits Tumor Growth in Head Neck Squamous Cell Carcinoma. *Annals of Translational Medicine* (2020) 8(6). doi: http://dx.doi.org/10.21037/atm.2020.02.145.
43. Patil S. Metformin Treatment Decreases the Expression of Cancer Stem Cell Marker Cd44 and Stemness Related Gene Expression in Primary Oral Cancer Cells. *Archives of Oral Biology* (2020) 113:104710.
44. Wang S, Wang J, Wang L. Metformin Inhibits Proliferation of Hypopharyngeal Carcinoma Cells by Regulating Mir-21-5p and Pdcd4 Expression. *Acta Medica Mediterranea* (2020) 36(3):1465-9. doi: http://dx.doi.org/10.19193/0393-6384_2020_3_228.

45. Zhang X, Dong Y, Zhao M, Ding L, Yang X, Jing Y, et al. Itgb2-Mediated Metabolic Switch in Cafs Promotes Oscc Proliferation by Oxidation of Nadh in Mitochondrial Oxidative Phosphorylation System. *Theranostics* (2020) 10(26):12044-59.
46. Hoppe-Seyler K, Herrmann AL, Däschle A, Kuhn BJ, Strobel TD, Lohrey C, et al. Effects of Metformin on the Virus/Host Cell Crosstalk in Human Papillomavirus-Positive Cancer Cells. *International Journal of Cancer* (2021) 149(5):1137-49. doi: https://doi.org/10.1002/ijc.33594.
47. Tsou YA, Chang WC, Lin CD, Chang RL, Tsai MH, Shih LC, et al. Metformin Increases Survival in Hypopharyngeal Cancer Patients with Diabetes Mellitus: Retrospective Cohort Study and Cell-Based Analysis. *Pharmaceuticals* (2021) 14(3):1-16. doi: http://dx.doi.org/10.3390/ph14030191.
48. Wei J, Huang J, Kuang Y, Li Y, Zhong D, Song J. Metformin Inhibits Proliferation of Oral Squamous Cell Carcinoma Cells by Suppressing Proteolysis of Nerve Growth Factor Receptor. *Archives of Oral Biology* (2021) 121:104971.
49. Yin W, Liu Y, Liu X, Ma X, Sun B, Yu Z. Metformin Inhibits Epithelial-Mesenchymal Transition of Oral Squamous Cell Carcinoma Via the Mtor/Hif-1alpha/Pkm2/Stat3 Pathway. *Oncology Letters* (2021) 21(1):31.
50. Chen X, Li C, Chen W, Lin S, Yi X, Lin Q, et al. Metformin Inhibits the Development of Hypopharyngeal Squamous Cell Carcinoma through Circ_0003214-Mediated Mir-489-3p-Adam10 Pathway. *Journal of Oncology* (2021) 2021 (no pagination).
51. Crist M, Yaniv B, Palackdharry S, Lehn MA, Medvedovic M, Stone T, et al. Metformin Increases Natural Killer Cell Functions in Head and Neck Squamous Cell Carcinoma through Cxcl1 Inhibition. *J Immunother Cancer* (2022) 10(11). Epub 2022/11/04. doi: 10.1136/jitc-2022-005632.
52. Liu S, Shi C, Hou X, Tian X, Li C, Ma X, et al. Transcriptional and H3k27ac Related Genome Profiles in Oral Squamous Cell Carcinoma Cells Treated with Metformin. *Journal of Cancer* (2022) 13(6):1859-70.
53. Zhang L, Sun Q, Ou Y, Zhang Q, Hu J. Metformin Induces Cytotoxicity in Oral Squamous Cell Carcinoma Cells by Targeting Ccn1/Akt-Axis. *International Journal of Pharmacology* (2022) 18(1):182-9.
54. Zhao W, Chen C, Zhou J, Chen X, Cai K, Shen M, et al. Inhibition of Autophagy Promotes the Anti-Tumor Effect of Metformin in Oral Squamous Cell Carcinoma. *Cancers (Basel)* (2022) 14(17). Epub 2022/09/10. doi: 10.3390/cancers14174185.
55. Ji M, Lv Y, Chen C, Xing D, Zhou C, Zhao J, et al. Metformin Inhibits Oral Squamous Cell Carcinoma Progression through Regulating Rna Alternative Splicing. *Life sciences* (2023) 315:121274. doi: https://dx.doi.org/10.1016/j.lfs.2022.121274.
56. Bairoch A. The Cellosaurus, a Cell-Line Knowledge Resource. *J Biomol Tech* (2018) 29(2):25-38. Epub 2018/05/29. doi: 10.7171/jbt.18-2902-002.
57. Crist M, Yaniv B, Palackdharry S, Lehn MA, Medvedovic M, Stone T, et al. Metformin Increases Natural Killer Cell Functions in Head and Neck Squamous Cell Carcinoma through Cxcl1 Inhibition. *Journal for immunotherapy of cancer* (2022) 10(11). doi: https://dx.doi.org/10.1136/jitc-2022-005632.
